# Structural Characterization of the Forensic Sodium Rhodizonate Test for Lead

**DOI:** 10.1021/acs.inorgchem.6c02536

**Published:** 2026-09-26

**Authors:** Alex Kukucka, Devin W. Thrush, Trent A. McHenry, Markéta Jarošová, Talaidh Isaacs, Anabelle Bahadur, Jan Rohlíček, Michelle Segura, Alexander G. Shtukenberg, Petr Brázda, Bart Kahr, Claudia E. Avalos

**Affiliations:** † Department of Chemistry and Molecular Design Institute, 5894 New York University, New York City, New York 10003, United States; ‡ Department of Structure Analysis, Institute of Physics, 86889 Czech Academy of Sciences, Prague 8 18200, Czech Republic

## Abstract

The forensic sodium rhodizonate test is used to detect lead in gunshot debris. Areas of interest are sprayed with a sodium rhodizonate solution followed by a tartrate buffer solution of pH 2.8, producing a deep red compound indicative of lead and other metals. A confirmatory hydrochloric acid spray produces a violet color specific to lead. The associated chemical transformations of the insoluble lead rhodizonate complexes have not been specified. Lead rhodizonate, lead rhodizonate tartrate, and lead rhodizonate chloride coordination polymers were synthesized from dilute solutions of sodium rhodizonate and lead nitrate in water, tartrate buffer, and HCl buffer. Nanoparticles of the precipitates were analyzed by three-dimensional electron diffraction (3D ED). A crystal, Pb_2_^4+^(C_6_O_6_)^2–^Cl_2_^2–^·3H_2_O, was characterized in the space group *R*3̅*m* (*a* = 8.0708(3) Å, *c* = 16.576(3) Å), with rhodizonate layers bidentately coordinated to three lead ions, offset by ca. 0.05 Å from the rhodizonate planes. Solid-state nuclear magnetic resonance revealed the presence of a single axial lead site in the nitrate precipitate, a distribution of lead sites in the lead rhodizonate tartrate precipitate, and three distinct sites in the lead rhodizonate chloride precipitate, suggesting other Pb compounds in the bulk coexisting with the 3D ED product.

## Graphical abstract

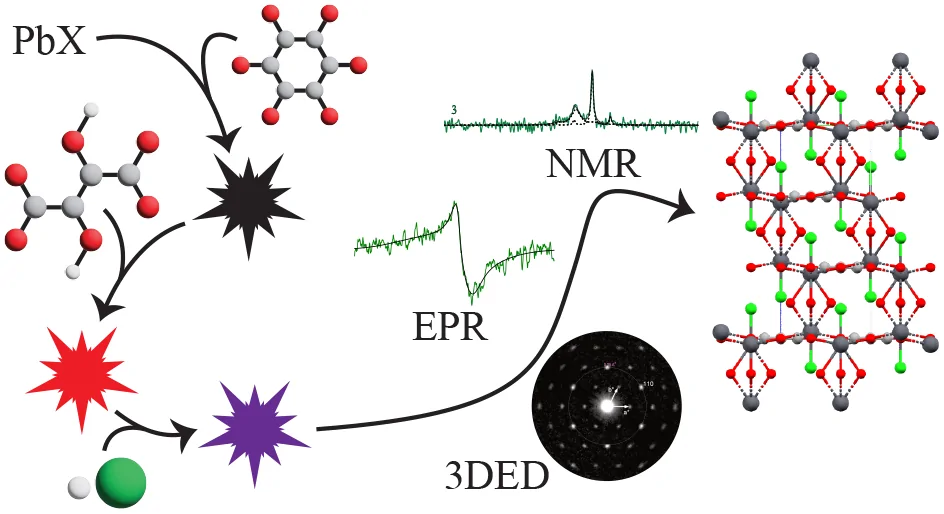

## 1 Introduction

Heavy-metal spot tests based on the reagent sodium rhodizonate^1^ were first described in 1942 ^2^ and gained popularity for the detection of lead in gunshot debris.^3^ A cloth exposed to gunshot residue, for example, is sprayed by an aqueous orange sodium rhodizonate solution, followed by a spray of tartrate buffer, resulting in a bright red color in the presence of lead­(II), among other heavy metals. Lead is distinguished by spraying with a 5% hydrochloric acid solution to produce a blue-violet color.^4^ This qualitative test is now widely available as sodium rhodizonate swabs for the detection of lead in drinking water, but they have been shown to be variable and equivocal.^5^ Forensic scientists have appreciated the swiftness of the rhodizonate test, enabling rapid evaluations on gunshot victims and potential perpetrators.^6^ However, sometimes the test meets fraught legal territory,^7^ due in part to the impermanence of the violet color of the final compound that is confirmatory of lead. Still, convictions and exonerations have been based on chemistry that we do not understand.

Silverman analyzed the compounds associated with the sodium rhodizonate test of lead.^8^ He synthesized an unreactive purple-black lead rhodizonate compound, a red lead rhodizonate tartrate compound from the tartrate buffer, and a violet lead rhodizonate chloride compound consistent with the forensic test after reacting with HCl solution. He proposed stoichiometries for these compounds based on X-ray photoelectron spectroscopy (XPS) and Fourier transform infrared spectroscopy (FT-IR): (**1**) lead rhodizonate [Pb_2_^4+^(C_6_O_6_)^2–^(OH)_2_^2–^(H_2_O)_5_], (**2**) lead rhodizonate tartrate [Pb_2_^4+^(C_6_O_6_)^2–^(C_4_H_4_O_6_)^2–^(H_2_O)_3_], and (**3**) lead rhodizonate chloride [Pb_3_^6+^(C_6_O_6_)_2_^4–^Cl_2_^2–^(H_2_O)_6_].^9^ Throughout, we will use the simple notation **1**, **2**, and **3** to indicate the sequential precipitates, irrespective of hydration states and stoichiometries. At least some of these materials are mixtures.

The introduction of more sensitive tests for environmental lead detection, based on the luminescence of lead-halide perovskites^10^ has been instrumental in forensic lead detection^11^ and in the detection of lead in paint.^12^ It is likely that these newer analyses will replace the reliance on the rhodizonate test. Future prospects notwithstanding, the rhodizonate test for lead remains a challenge for structural chemists.

**1** was synthesized from dilute solutions of lead nitrate and sodium rhodizonate. Complexes **2** and **3** were made by the sequential addition of tartrate and HCl buffers, respectively, according to Silverman.^8^ The resulting precipitates were analyzed by powder X-ray diffraction (PXRD), FT-IR, UV–vis spectroscopy, solid-state NMR (^207^Pb, ^13^C, ^1^H), electron paramagnetic resonance (EPR), and, most saliently, by three-dimensional electron diffraction (3D ED) for small-molecule structure determination. In this way, the crystal structure of **3** was determined. For those compounds not amenable to single-crystal structural analysis, solid-state ^207^Pb NMR chemical shifts followed local structural changes at Pb sites throughout the rhodizonate test process, even for materials that could not be characterized by diffraction. While the structure obtained from electron diffraction and the resulting solid-state NMR results does not wholly narrate all the products in the sodium rhodizonate test for lead, it illustrates the terminal product of the sequence of reactions in the spot test as well as local structural changes across the sequence.

As we were completing this manuscript, a multipronged approach to the structural chemistry and colorimetry of the metal rhodizonate test was published by Hopkins and coworkers (hereafter Hopkins).^13^ They have well illustrated the chemical complexity of the rhodizonate test and report eight single-crystal structures of metal complexes that arise. There are some important differences between the two studies. Hopkins studied Pb^2+^ and Ba^2+^ complexes. We only studied Pb coordination.^13^ Their source of Ba^2+^ was barium nitrate and their source of Pb^2+^ was lead acetate trihydrate or lead nitrate. Three of the eight structures are oxalate complexes even though no oxalate was added. Oxalate is presumed to arise from decomposition of rhodizonate to croconate, followed by oxidation. Decomposition products—croconate, oxalate, and a croconate dimer-derived chromophore—have time to accumulate in the long crystallization times by counter-diffusion of metal salts and sodium rhodizonate that were employed by Hopkins.^13^ We studied the instantaneous precipitates of coordination polymers as they seemed to best mimic the rhodizonate spot test in forensics. Lastly, while Hopkins studied the first steps in the precipitation process, we focused on the last step. The first steps present a red spot test characteristic of a divalent metal. The last step discriminates lead from other metals, as the stain becomes purple after spraying with HCl. Hopkins described coordination polymers of lead rhodizonate as “elusive″.^13^ We provide one herein.

## 2 Results

### 2.1 X-ray and Electron Diffraction

**1**, **2**, and **3** were subject to PXRD and 3D ED analyses. Nanocrystals of **3** were stable in the electron beam and provided good quality diffraction patterns, with a maximum usable resolution of 1.6 Å^–1^ for most crystals probed. The lattice parameters were *a* = 8.0708(3) Å, *c* = 16.576(3) Å. An image of the grain size and morphology is shown in Figure S1. Impurities were also observed in the sample, as shown in Table S2. The most abundant were crystals of PbCl_2_ that were too large to be measured. Dynamical refinement^14^ of the structure of **3** was done in *R*3̅*m* space group, and the *R*_*obs*_ and *wR*_*all*_ were 5.32 and 5.91%, respectively. The positions of hydrogen atoms were found in the difference potential map and refined without any restrictions. The structure was deposited to the Cambridge Crystallographic Data Centre (CCDC) under deposition number 2523467.

As shown in Figure 1, the structure of **3** consists of near planar layers of rhodizonate ions, each coordinated to three Pb ions in a bidentate fashion to make trigonal networks (Figure 1a). The Pb ions and rhodizonate rings sit on trigonal axes. An axial chloride ligand is directed toward the rhodizonate ring centroid in an adjacent layer. This distance is just 3.22 Å^15^ suggesting an unusually close anion−π interaction.^16^ The directional chloride ligands are balanced on the other side of the half sphere of lead by three water molecules forming a triangle. There is statistical disorder of the water molecules about a pseudo-6-fold axis. The coordination number of lead is ten. The 12 rhodizonate ring atoms (6 oxygens and 6 carbons) all lie in a common plane. Hydrogen atoms form hydrogen bonds with neighboring chloride atoms. **3** was prepared and filtered to remove any excess PbCl_2_ and PXRD was measured. The PXRD of **3** shown in S3, confirms the presence of the phase observed by 3D ED. The lattice parameters using Le Bail fit refinement were *a* = 8.05(1) Å and *c* = 16.7(1) Å. Another crystalline phase was present in the sample following PbCl_2_ removal, and the positions of the extra peaks did not correspond to PbCl_2_. This unknown phase was not present prior to PbCl_2_ removal.

**Figure 1:**
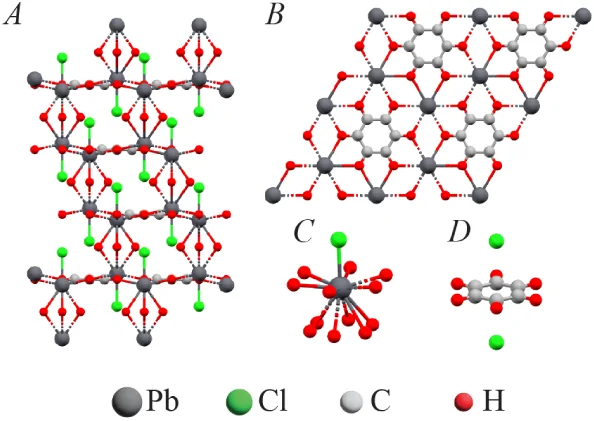
3D ED structure of **3**. (A) Near-planar layers of rhodizonate ions and lead ions; (B) view along the trigonal axis showing layers of rhodizonate ions with bidentate coordination to lead; (C) coordination geometry of lead with a holodirected ligand sphere. The oxygen atoms in the bottom plane have only half occupancy; (D) view of the axial chloride ligand directed toward the rhodizonate ring.

Crystals of **2** diffracted poorly, with a maximum resolution below 0.5 Å^–1^. They gave primitive hexagonal lattice parameters of *a* = 7.97(3) Å, *c* = 13.80(9) Å (Table S1). It was not possible to determine the Laue class due to the low quality of the diffraction data. The lattice parameter *a* is similar to that of **3**. PXRD of **2** is dominated by a pattern that can be indexed with a hexagonal cell with unit-cell parameters *a* = 8.084(5) Å and *c* = 13.64(5) Å, which matches very well with the lattice parameters from 3D ED. A minor unknown impurity was also present in the diffractogram (Figure S5). Assuming that the backbone of **2** and **3** are the same (layers of sheets of Pb_2_^+2^(C_6_O_6_)^2–^) a reasonable model may be constructed from ED data. Reflections {110} in the *hk*0 reciprocal-space section (Figure S7A) are significantly more intense than the other reflections. This suggests that the distance between the lead atoms is about 4.6 Å. Reflections in the 0*kl* reciprocal-space section (Figure S7B) show that the *hkl* reflections with odd *l* are much weaker than those with *l* even. This indicates that the two halves of the unit cell (divided by a plane in the middle of the c lattice parameter) are very similar. Two structural models (Figure S8) for **2** are proposed, one with layer sequence AAAA (Model 1), the other with ABAB (Model 2). The layer sequence in **3** is ABCABC. Tested against powder diffraction data, the match was reasonable for both models. It is not possible to determine whether lead is in the plane of the layer or out of it for **2**.

The distance between two rhodizonate layers in **3** is 5.57 Å, while it is 6.82 Å in **2**. In **3,** the interlayer space is occupied by three water molecules and a chlorine atom. These species have a combined volume of ∼75 Å^3^. The space between the layers in **2** is larger by about 71 Å^3^ than in **3**. This leads to an interlayer volume of 146 Å^3^ in **2**. The estimated volume of the tartaric acid in a crystal structure^17^ is about 154 Å^3^ which matches well with the predicted void between the lead rhodizonate layers in **2**. It cannot be excluded that there are a few water molecules between the lead rhodizonate layers. If the lead rhodizonate sheet is perfectly ordered, then it is likely that the tartaric acid is highly disordered and causes poor crystallinity of the material.

The 3D ED of **1** did not reveal any crystalline material. PXRD revealed a pattern that was successfully fitted by a Le Bail fit using lead oxalate lattice parameters.^18^ The profiles were rather broad, and the width was different for different reflections. The sample contains a considerable amount of amorphous material, with a minor crystalline component. The 3D ED and PXRD results are in good agreement that **1** is amorphous and that lead oxalate is only a minor impurity.

We also measured **1, 2,** and 3 nine months from synthesis (Figures S9, S10, S11). **3** decomposes to lead oxalate, and any minor peaks could not be indexed. Over time, **2** converts into several crystalline phases, and the structure identified in the fresh sample may still be present. **1** contains lead oxalate and a substantial amount of an amorphous material.

### 2.2 Solid-State NMR

Lead­(II) rhodizonate (**1**), lead­(II) rhodizonate/tartrate (**2**), and filtered lead­(II) rhodizonate/chloride (**3**) were analyzed by solid-state NMR. While precipitates **1** and **2** were prepared in an identical way as those analyzed for X-ray diffraction and 3D ED, precipitate **2** was filtered and placed in a fresh tartrate buffer solution prior to forming precipitate **3** in order to remove any excess PbCl_2_.

#### 2.2.1 ^207^Pb

^207^Pb NMR chemical shifts are particularly sensitive to coordination.^19–21^ In the Herzfeld–Berger convention^22^ δ_*iso*_ is the isotropic value of the chemical shift tensor (=(δ_11_ + δ_22_ + δ_33_)/3), Ω is the span (=δ_11_ - δ_33_ (Ω ≥ 0)), and κ is the skew (=3­(δ_22_ - δ_*iso*_)/Ω, (−1 ≤ κ ≤ 1)). A larger Ω is characteristic of greater electron-density anisotropy. κ, the skew, characterizes the symmetry of the chemical shift tensor as either axial (κ = ± 1), isotropic (κ = 0), or rhombic (κ ≠ 0, κ ≠ ± 1). δ_11_, δ_22_, and δ_33_ are the principal components of the chemical shift tensor according to IUPAC conventions, where δ_11_ (δ_33_) corresponds to the direction of least (highest) shielding.^23^

The observed chemical shifts in this series fall within the range typical of ionic ^207^Pb compounds.^24^ ^207^Pb NMR of **1** shows a site with δ_*iso*_ = −1658.0 ppm, Ω = 1596.0 ppm, and κ = 1, consistent with axial symmetry and electron density perpendicular to the plane of the principal axis (cigar-like). If we compare the lead NMR of **1** to that of lead oxalate, shown in Figure S12, as well as the fits obtained in Table S3 the parameters are nearly identical, consistent with the PXRD results showing the presence of lead oxalate in **1**. Measurements taken at a shorter echo time (166 μs) indicate that a broad component centered about −1655.5 ppm is also present (Figure 2a). The broader component is characterized by inhomogeneous Gaussian broadening and a shorter $T_{2}^{*}$ , consistent with positional disorder at the lead site.^25^

**Figure 2:**
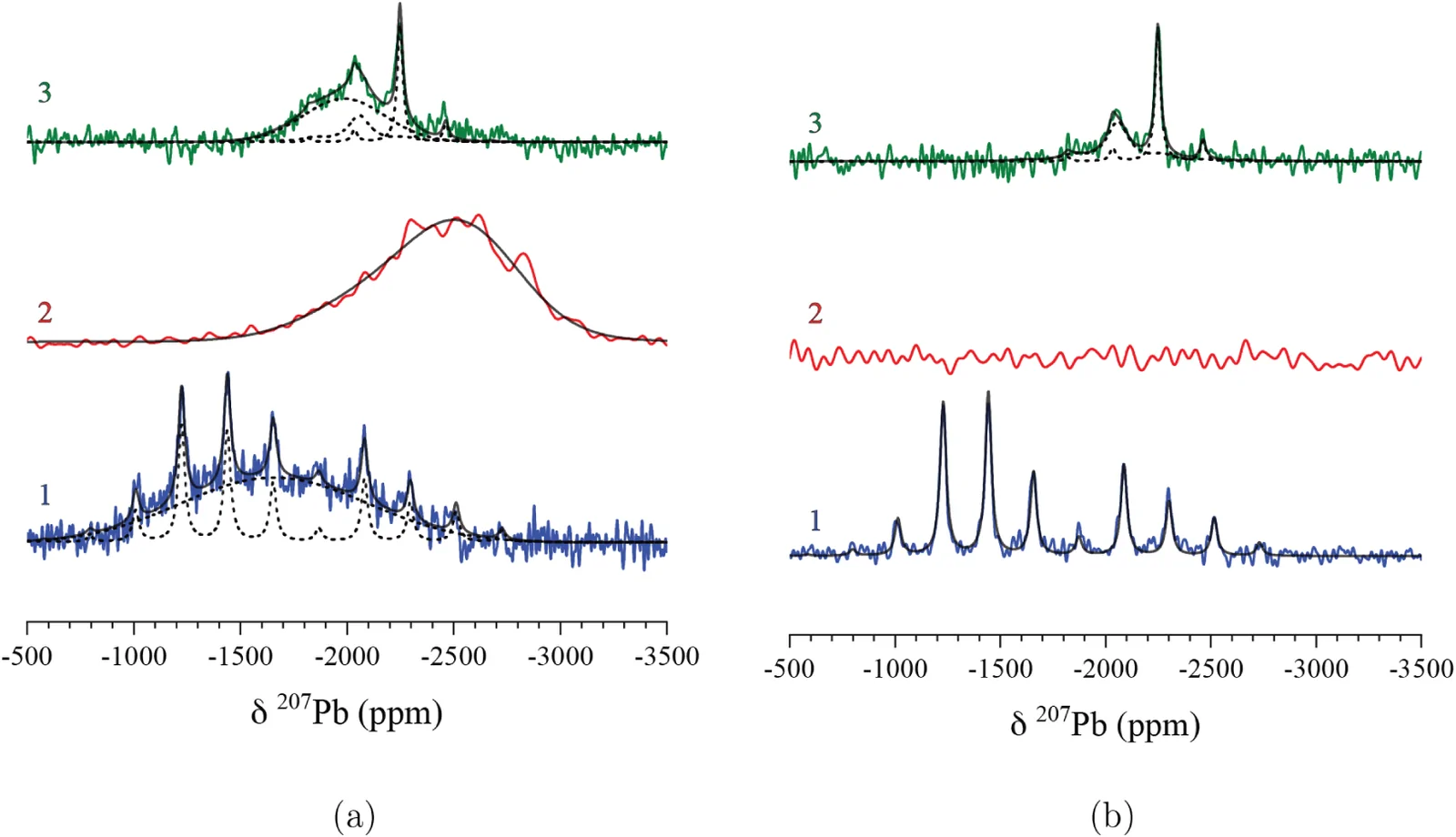
(a) ^207^Pb NMR echo measurements of **1** (blue), **2** (red), and **3** (green) taken at 275 K at 18 kHz MAS, with an echo delay of τ = 166 μs; (b) τ = 555 μs.

**2** is more shielded (δ_*iso*_ = −2423.8 ppm) with a smaller Ω = 908.0 ppm and κ = −1. It shares the axial character of **1**, although the skew is reversed. A change in the sign of κ indicates a change in the local electron density distribution from prolate (κ = +1) to oblate (κ = −1). In **2**, no signal is detected using a longer echo delay, indicating a shorter spin–spin relaxation time compared to **1** and **3**. **2** also exhibits a spin–lattice relaxation rate (39 ms) that is orders of magnitude faster than **1** (7.0 s) and **3** (3.2 s). **3** shows two distinct sites with isotropic chemical shifts of δ_*iso*_ = −2057.6 and δ_*iso*_ = −2248.3 ppm. Both peaks have smaller chemical shift anisotropy (CSA) than the lead sites present in **1** and **2**, as evidenced by the reduced intensity of sidebands. The reduced CSA suggests a more electronically symmetric lead site in the chloride structure.^19^ As in **1**, a shorter echo delay reveals a broad component centered at −1969.0 ppm.

Correlations among lead–oxygen coordination geometries, bond lengths, and chemical shifts have been previously established in a series of lead oxides.^19^ Longer lead–oxygen bond lengths and higher coordination numbers (CN) are shielding, while shorter bonds are deshielding. **1** is the most deshielded, while **2** is the least. This could be related to the difference in CN, given that the presence of tartrate would likely alter the number of coordinating oxygen atoms.

In addition, the reduced *T*_1_ and spectral broadening in **2,** as compared to **1** and **3** (Figure 2) are consistent with a change in molecular dynamics or possibly paramagnetic relaxation enhancement; **2** had a five times larger EPR signal compared to **1** and **3**.

#### 2.2.2 ^13^C NMR

Direct ^13^C and cross-polarization (CP) 1D NMR measurements were performed on all three rhodizonate precipitates. In a CP experiment, spin magnetization is transferred between ^1^H and ^13^C spin systems via the dipolar interaction, which depends on the distance (1/*r* ^3^) between the two nuclei. Direct measurements are shown in Figure 3 while CP measurements are shown in Figure S14.

**Figure 3:**
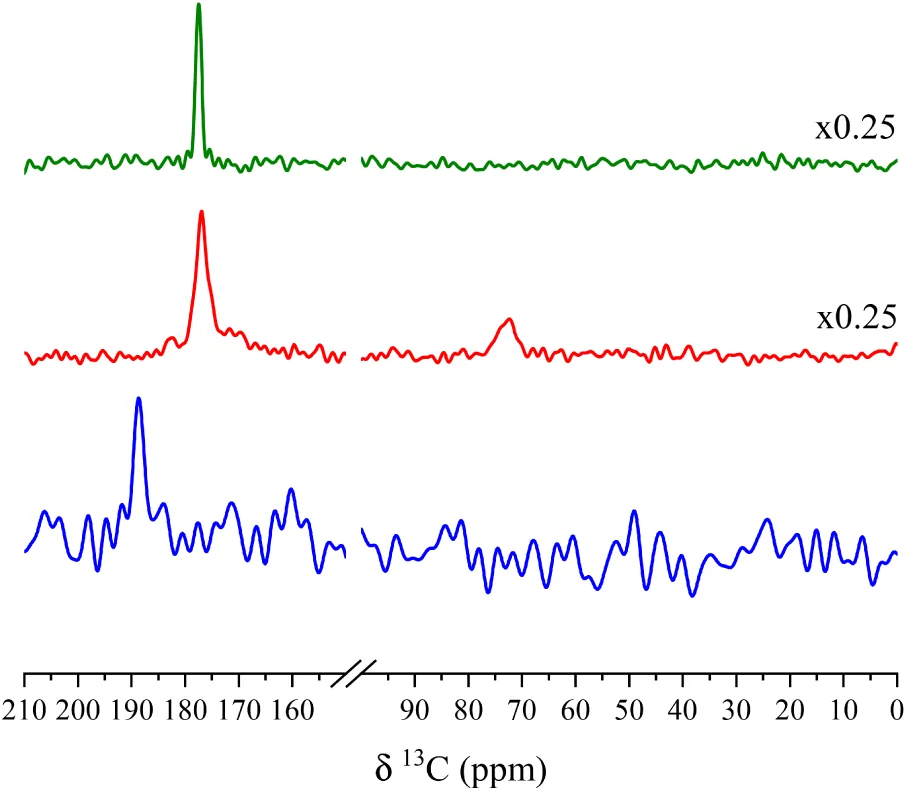
^13^C NMR echo measurements of **1** (blue), **2** (red), and **3** (green) at 18 kHz MAS.

The rhodizonate peak was found at 181.3 ppm in the starting material, sodium rhodizonate. A single carbon signal is observed from precipitate **1**. This site is more deshielded, with a chemical shift of 188.7 ppm. The carbon chemical shifts on oxocarbons are very sensitive to their environment; changes in solvent used in liquid-state NMR experiments can lead to shifts of nearly 10 ppm.^26^ FTIR spectra of **1** (Figure S2) show changes in the C–O stretching frequency consistent with bidentate coordination of a metal to rhodizonate or a related oxocarbon complex. Bidentate binding leads to a sawtooth pattern at 1080 and 1062 cm^–1^.^27^ This coordination could result in deshielding of the carbon in **1** as compared to sodium rhodizonate. No ^13^C CP signal is observed from **1** consistent with remote or highly mobile protons (Figure S14).

**2** shows a peak at 176.9 ppm corresponding to the rhodizonate carbon and another at 72.4 ppm that can be attributed to the tartrate. In **3**, only the rhodizonate carbon at 177.5 ppm is observed, as expected. This is supported by the FTIR results (Figure S2). Our results may be compared to fully deprotonated aqueous rhodizonic acid, which is observed at 177.9 ppm.^28^ **1** has the largest chemical shift, suggesting a stronger ketone (less aromatic) character of the C–O bond. The recovery of a more shielded lead site in **2** and **3** then may suggest a reduced interaction between the lead and the rhodizonate carbon or could be due to the decomposition of lead rhodizonate to lead croconate in precipitate **1**. Aside from **1**, all CP and direct measurements show similar results, consistent with the spatial proximity of ^1^H nuclei to the rhodizonate carbon. We propose that coordinated water likely contributes to the observed CP signal in **3**, while in **2** tartrate hydroxyl groups may also participate.

#### 2.2.3 ^1^H NMR

^1^H NMR spectra are shown in Figure 4, with fitting parameters and chemical shift and line-width errors are provided in Table S5. **1** shows a single peak at 4.5 ppm. FTIR of **1** suggests either a bound hydroxyl or hydrogen-bonded water, indicated by a weak, broad band at 3335 cm^–1^ (Figure S2). Hydroxides exhibit shielded protons, with shifts ranging from 1.4 ppm to <0 ppm, whereas hydrates are often observed around 5.0 ppm. The chemical shifts, line widths, and the appearance of MAS sidebands are characteristic of bound water (hydrates) rather than higher-mobility water.^29^

**Figure 4:**
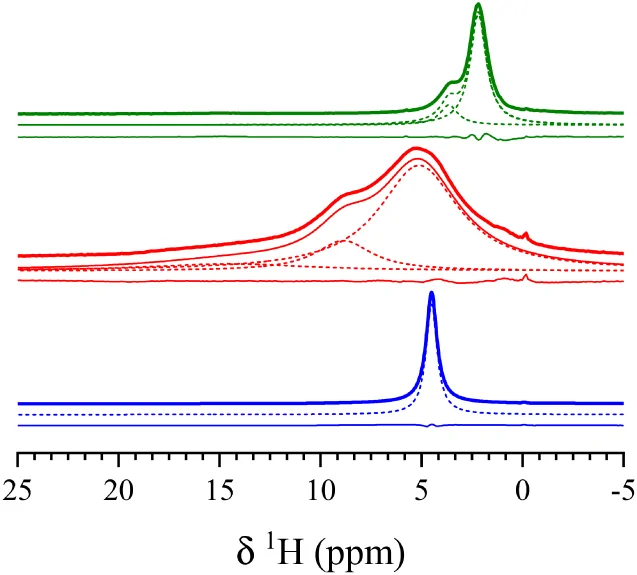
^1^H NMR echo of **1** (blue), **2** (red), and **3** (green) at 275 K at 20 kHz MAS. Fit components (dashed) and experimental data (solid) are shown along with residuals.

**2** exhibits peaks at 5.1, 8.8, and 14.5 ppm that are considerably broader than those from **1** or **3**. The peak at 5.1 ppm corresponds to the α-protons of the alcohol and hydroxyl groups, while that at 8.8 ppm likely corresponds to protons adjacent to an aromatic moiety, possibly due to weak OH−π interactions with the rhodizonate ring.^30^ The FTIR spectrum of **2** shows a broad, medium-intensity peak at 3178 cm^–1^, consistent with a coordinated hydroxyl. In **3**, two peaks are observed, one at 2.1 ppm associated with an R–OH group and another at 3.6 ppm, which could arise from bound water.

### 2.3 Continuous-Wave Electron Paramagnetic Resonance

CWEPR of **1**, **2,** and **3** were measured at 60 K (Figures 5 and S18) and 297 K (Figures S19, S20). EPR signals with a *g*-factor of ∼2 were observed in all precipitates. No ^207^Pb hyperfine was observed. We attribute the EPR signal to an oxocarbon-centered radical distributed about the rhodizonate ring in **1, 2,** and **3**.^31,32^ Fitting parameters for spectra taken at 60 K are shown in Table S9. The average *g*-factor, *g*_*iso*_, is useful for determining the radical present in cyclical oxocarbons such as rhodizonate.

**Figure 5:**
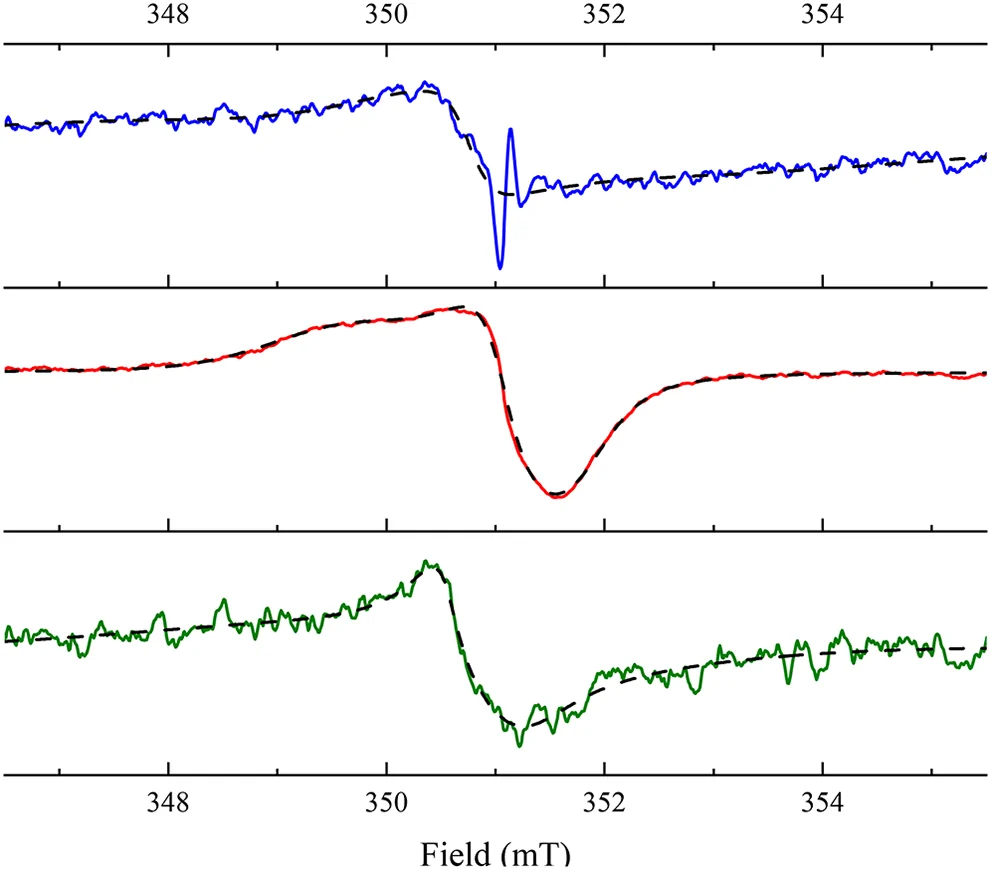
X-band EPR measurements taken at 60 K and plotted at 9.85 GHz for **1** (blue), **2** (red), and **3** (green), with respective fits (dashed lines).

EPR spectra of **1** and **3** are fit to two components. Values extracted from two-component fits were more consistent with recorded rhodizonate radical *g*-factors than single-component fits (Δ*g* < 0.009). The *g*-factor for oxocarbon radicals has been observed to decrease when bound to Pb^33,34^ to yield an EPR signal that is the sum of both bound and unbound cases. Hence, we consider the C_6_O_6_^3–^ trianion radical in bound and unbound forms to tentatively assign the observed EPR spectra. Precipitates **1** and **3** contain radicals with *g*_*iso*_ = 2.0048 and 2.0053, respectively, labeled as **1**’ and **3**′ in Table S9. This *g*_*iso*_ is comparable to the *g*-factor that has been reported for the radical trianion, C_6_O_6_^3–^, in water (*g*_*iso*_ = 2.00487), and for the surface-adsorbed radical trianion (*g*_*iso*_ = 2.00457).^31,35^ The radical density is localized on the oxygen with a smaller spin density distributed on the carbon atoms. However, the presence of a radical trianion species is surprising given the mild synthesis conditions. In addition, the “unbound” signal is significantly broader than the bound form, consistent with different spin relaxation behavior. Such broad signals could also be characteristic of aggregation or radicals in amorphous structures. The dominant EPR signal in **1**, **2**, and **3** arises from a radical species with significantly smaller *g*-factors of *g*_*iso*_ = 1.9970, 2.0025, and 2.0024, respectively. We also attribute this radical to a carbon-centered radical on the rhodizonate.^32^ Bidentate binding of lead to oxygen substituents in phenol radicals (e.g., gallic acid) leads to a lower *g*-factor relative to the unbound form due to an increase in spin density on the carbon atoms in the ring.^33^ Likewise, coordination of lead to the trianionic rhodizonate species would lead to a reduction in the observed *g*-factor. Oxidation of a rhodizonate salt produces a monoradical C_6_O_6_^•–^ which has a *g*-factor of 2.00652;^32^ this is larger than the *g*-factor we measure. More accurate *g*-factors and more confident assignments could be obtained at higher magnetic fields where all components of *g* are better resolved.

We could attribute the higher *g*-factor species (**1**’ and **3**’) to oxocarbon radicals that are not coordinated to lead, and so, are adjacent to a lead vacancy in the structure. The *g*_*iso*_ = 2.00487 radical is similar to the *g*_*iso*_ for sodium rhodizonate as well as for that of unbound gallic acid,^33,34^ consistent with less spin density on the carbons and more on oxygen. This species is not readily observed in **2,** as it is obscured by the larger signal.

EPR spectra for all lead precipitates are more anisotropic than those of the starting material, sodium rhodizonate. The sodium rhodizonate radical exhibits near-axial *g*-anisotropy, while **1**, **2**, and **3** have rhombic *g*-tensors. The differences in line shape between **1** and **2** indicate that the tartrate likely interacts with the rhodizonate radical, perturbing its local environment. Further discussion about changes in the *g*-factor and coordination is included in the Supporting Information.

## 3 Discussion

3D ED and PXRD provide a structure for a component of **3,** and lattice parameters with model structures for **2**. **1** was found to be amorphous, with crystalline lead oxalate as a minor component.

Here, we discuss these results in light of our magnetic resonance data. **1** contains a structure with a single axial lead site and one with significantly more positional disorder. The electron density about both of the lead sites is prolate, with greater shielding likely perpendicular to the oxocarbon plane. Such hemidirected coordination geometries are typical of Pb­(II) coordination polymers.^36,37^ Based on the significant differences in ^207^Pb and ^13^C chemical shielding between **1** and **2, 3**, it can be inferred that the lead–oxygen bonds of the crystalline and amorphous components of **1** are shorter than those in **2** and **3**. The isotropic chemical shift of the crystalline ^207^Pb site in **1** is nearly identical to that of lead oxalate, which also has a hemidirected lead site,^18^ while that of the amorphous component is more deshielded. The ^13^C and ^1^H NMR of a fresh sample of **1** do not solely match that of lead oxalate, consistent with the presence of a different structure (Figure S12a,b). From the EPR results, **1** tentatively contains both lead-coordinated and nonlead-coordinated oxocarbon radicals. The observed radicals have a rhombic *g*-tensor typical of rhodizonate-type radicals, both of which are present at low concentrations.

Considering these results, along with the PXRD data, **1** likely consists of lead oxalate as well as another structure with a hemidirected Pb­(II) coordination geometry. This would explain the observed rhombic *g*-tensors of the rhodizonate radicals, as well as the axial shielding at the lead site. The ^1^H chemical shifts of **1,** along with the absence of a CP signal indicate that the water present in these structures is bound and remote from the oxocarbons in the material. One of the lead sites in **1** likely exhibits a considerable amount of positional disorder, leading to the observed amorphous structure observed in 3D ED and PXRD.

Crystals of **2** have a single axial lead site with a larger CN than structures in **1, 3**. The asymmetry in the electron density about the lead site is reduced and changes from prolate to oblate, consistent with tartrate coordination between the rhodizonate layers. Electron density shifts away from the ring to the interspace layer as ^13^C chemical shifts of the rhodizonate carbons in **2** match that of unbound rhodizonate in a liquid. The rhodizonate-centered radical has a rhombic *g*-tensor, with the average reflecting a value consistent with rhodizonate coordinated to lead. Protons associated with tartrate are close to the rhodizonate ring and may form weak OH−π bonding interactions. The distribution of ^1^H shifts indicate that chemical disorder is introduced with the addition of tartaric acid and is subsequently removed upon addition of HCl as seen in the ^1^H NMR of **3**. This observation is consistent with structural models of **2** from 3D ED and PXRD, which suggest a disordered tartaric acid component likely leads to poor crystallinity in **2**.

From 3D ED, **3** contains near planar layers of rhodizonate ions and a 10-CN lead site ca. 0.05 Å from the rhodizonate plane. From NMR, three lead sites are present in the bulk precipitate of **3**. One exhibits considerable positional disorder, while the other two show a single site with reduced shielding anisotropy relative to **1** and **2**, consistent with more symmetric electron density. Two different ^1^H environments are observed, which we attribute to coordinated water. 3D ED shows the presence of water molecules as integral to the structure with partial occupation. Differences in CN can have a significant effect on chemical shifts; therefore, one of the lead sites likely has a different CN and water coordination environment than the other. Rhodizonate coordinates in a bidentate fashion to three lead ions, and the chloride in close proximity to the rhodizonate ring indicates anion-π interactions,^16,38^ albeit there is little evidence of this interaction in the magnetic resonance data. The observed oxocarbon radical is nearly axial in **3**, while it is found to be more rhombic in **1** and **2**, which may point to some contribution of the Cl in shaping the unpaired electron spin density on the rhodizonate (Table S9).

What causes the observed color change in the three precipitates, **1** (dark purple, red-shifted), **2** (red, blue-shifted), and **3** (violet, most red-shifted)? The rhodizonate ion in water, C_6_O_6_^2–^, has a strong absorption in the blue and appears orange. This absorption will shift in the presence of a counterion and is red-shifted when bound as Li_2_C_6_O_6_.^39^ In **1,** the lead sits above the plane of rhodizonates and the electron density on the rhodizonate is likely rhombic; in **2,** the lead is coordinated to the tartrate, which also coordinates to the rhodizonate ring via OH−π interactions; in **3,** the lead is coordinated to the chlorine, which forms a weak anion-π interaction. Following several months of “shelf-storage” in yttrium-stabilized zirconia solid-state NMR rotors, NMR measurements on all three samples were repeated, and the resulting evolution can be observed in Figure S17. By eye, the color of all three samples did not noticeably evolve over this period; however, based on the NMR results, the chemistry has clearly changed. We observe increased formation of lead oxalate in **1** and **3,** as was observed in samples prepared by Hopkins.^13^

Photosensitivity and hydration may contribute to the long-term stability of these solids. Solid forms of dianionic rhodizonate, such as sodium rhodizonate, are generally regarded as stable, even when stored in oxygenated environments and exposed to visible and UV light,^8,40^ while aqueous rhodizonate species exhibit considerable photosensitivity and decay on the order of hours to days into croconate and oxalate products, depending on solvent conditions.^8^ PXRD of degraded **3** shows the presence of an oxalate degradation product. 3D ED and solid-state NMR support the presence of water of hydration in close proximity to the rhodizonate rings (3.311 Å) in the major structure of **3** prior to degradation. These waters of hydration could provide a route for photodegradation of **3**. Sodium rhodizonate has high photostability^40^ and exhibits an EPR signal with a *g*_*iso*_ of 2.0046 (Table S9), comparable to that of rhodizonate (*g*_*iso*_ of 2.00487 and 2.00457)^31,35^ and *g*_*iso*_ measured for **1**’ and **3**’. Croconate exhibits a measurably distinct *g*_*iso*_ of 2.00624 ^31^ and was not observed in any of the EPR measurements. However, it is possible that the observed broad EPR signals could obscure this minor decay product. Clearly, correlating structural models to the observed color changes would require further sample purification, controlled investigation of photodegradation products, as well as a more thorough analysis of electronic structure using time-dependent density functional theory.

## 4 Experimental Section

### 4.1 Synthesis

#### 4.1.1 Lead­(II) Rhodizonate: **1**

Lead­(II) rhodizonate was synthesized by mixing 64.2 mg Na_2_C_6_O_6_ (sodium rhodizonate, BeanTown Chemical) in 100 mL of distilled, deioinized water (3.00 mM) with 99.4 mg of Pb­(NO_3_)_2_ (lead­(II) nitrate, Sigma-Aldrich) in 100 mL of water (3.00 mM), yielding a 200 mL total reaction mixture. A dark purple-black precipitate formed immediately, which was vacuum filtered on a fritted filter (4–5.5 μm pores) with water washes and left to dry overnight with laboratory lights turned off. When dried, this compound was scraped from the filter and stored in an airtight glass vial for further analyses. FT-IR (cm^–1^): 3335 (w,br), 1572 (s), 1474 (s), 1367 (s), 1286 (s), 1080 (w), 1062 (m), 771 (s, sh), 590 (m).

#### 4.1.2 Lead­(II) Rhodizonate Tartrate: **2**

A tartrate buffer of pH 2.8 was created by dissolving 660 mg of *L*-tartaric acid (Amresco) and 860 mg of potassium *L*-tartrate (Sigma-Aldrich) in 100.00 mL of water. To speed up the dissolution process, this solution was heated to 60^◦^C while stirring at 200 rpm for 5 min. After forming the buffer, 535.1 mg of Na_2_C_6_O_6_ was dissolved directly to the buffer solution to make a 0.025 M solution of sodium rhodizonate. 828.0 mg of Pb_2_(NO_3_)_2_ was then dissolved directly into this solution (a 1:1 molar ratio to sodium rhodizonate), forming a bright red precipitate upon combination. The precipitate was vacuum filtered on a fritted filter (4–5.5 μm pores) with water washes, and dried overnight with the laboratory lights turned off. After drying, this compound was removed from the filter and stored in an airtight glass vial for further analyses, and used in the synthesis of lead rhodizonate chloride. FT-IR (cm^–1^): 3178 (m, br), 1630 (w), 1575 (m), 1530 (m), 1450 (vs), 1421 (vs), 1306 (m), 1260 (m), 1180 (w), 1114 (s, sh), 1069 (s, sh), 1049 (m), 1000 (vw), 925 (vw), 900 (vw), 837 (w), 810 (w), 772 (m), 715 (m), 650 (w), 570 (2).

#### 4.1.3 Lead­(II) Rhodizonate Chloride: **3**

To replicate the conditions of the forensic sodium rhodizonate test in which hydrochloric acid is added to the red compound in tartrate buffer, a new pH 2.8 tartrate buffer was created. The p*K*_*a*_ of rhodizonate is 4.2. 50.00 mL of tartrate buffer was made by dissolving 330 mg of L-tartaric acid and 430 mg of potassium L-tartrate in 50.00 mL of water. Dissolution was facilitated by heating the solution to 60^◦^C at 200 rpm for 5 min. 400 mg of the red lead rhodizonate tartrate compound was added to the tartrate buffer solution. 50 μL of concentrated HCl (Fisher Chemical) was added to this solution, then thoroughly swirled to combine, forming a violet precipitate. The precipitate was vacuum-filtered on a fritted filter (4–5.5 μm pores) with water washes, and dried for 3–4 h to limit air exposure. After drying, the compound was stored in an airtight glass vial at 4^◦^C. Although this procedure successfully yielded a compound with the anticipated violet color, a yellow decomposition product characteristic of croconate^8^ could be detected if the compound was dried slowly for extended periods over 4 h. Samples used for PXRD, 3D ED, and NMR analysis were selected on the basis of lacking visible yellow decomposition product and were dried over a shorter period of time. Vacuum filtration should be performed immediately after precipitate formation to minimize the extent of decomposition. FT-IR (cm^–1^): 3365 (m), 1725 (w), 1679 (w, sh), 1629 (w, sh), 1421 (vs), 1300 (m), 1090 (m), 1070 (m), 944 (m), 810 (m), 714 (m), 614 (m).

Given the photosensitivity of rhodizonate compounds in solution, several measures were taken to limit their time in solution as well as their exposure to light during the synthesis of **1**, **2**, and **3**. This included preparation and handling in amber vials and maintaining a low-light environment by keeping the fume hood light off. In the preparation of precipitates **2** and **3**, the use of tartrate buffer solution (pH 2.8) has been reported to stabilize the rhodizonate anion.^41^ Although light exposure was limited during routine handling, photosensitivity was not being investigated as an independent variable; thus, exposure was not systematically controlled or quantified. Following preparation, all samples were stored in the solid phase and in dark.

Samples were immediately measured following synthesis. While rhodizonate is known to photodegrade rapidly in the presence of light when dissolved in solution, we did not observe such fast degradation in the solid state over the course of our experiments, though long-term degradation was observed and later discussed. Comparisons of NMR and EPR spectra taken weeks apart showed no changes. All lead-containing samples were always handled with gloves and disposed of in clearly labeled waste containers.

### 4.2 Solid-State NMR

Samples **1**, **2**, and **3** were stored and sealed at atmospheric conditions in glass vials with plastic screw caps prior to measurement. **3** was likewise stored in a glass vial with a plastic screw cap, but was kept in a −20^◦^C fridge. Solid-state NMR experiments were carried out on a 3.2 mm CPMAS DNP probe at 400 MHz with an AVANCE NEO console. All spectra were collected at 275 K using a BCU II temperature controller. All ^207^Pb NMR measurements were referenced to lead nitrate at 295 K with δ_*ref*_ = −3490 ppm with respect to TMS.^42^ A standard echo sequence was used, and full or half echoes were collected for all ^207^Pb measurements, using 500- and 100-μs windows depending on the $T_{2}^{*}$ parameter. A recycle delay of 32 s was used for **1** and **3,** while a relaxation delay of 0.4 s was used for lead rhodizonate tartrate. Measurements were conducted at multiple spinning speeds to assess the presence of multiple sites in **3**. ^1^H and ^13^C measurements were also collected on all three samples using a standard echo sequence, and half echoes were collected using an echo delay of one rotor period. Cross-polarization measurements were also taken of all three samples using RF power of 83 kHz for ^13^C and 69 kHz for ^1^H, with a contact time of 9000 μs at 10 kHz MAS. A recycle delay of 1 s was used for ^1^H echo and ^13^C CP NMR measurements, while a recycle delay of 13 s was used for ^13^C measurements. ^13^C NMR measurements were collected using a standard decoupling sequence, SPINAL-64. ^1^H and ^13^C spectra were referenced to adamantane (^1^H δ_*ref*_ = 1.91 ppm^43^ and ^13^C δ_*ref*_ = 37.778 ppm with respect to TMS^43^ for the methine group). Full experimental details regarding number of scans, RF powers, and recycle delays are included in the Supporting Information. Isotropic and anisotropic components were extracted by fitting of 1D echo spectra using SOLA on TopSpin. Spin relaxation measurements on ^207^Pb and^1^H were collected using a standard saturation recovery sequence and fit as described in the Supporting Information.

### 4.3 Electron Paramagnetic Resonance

Continuous-wave EPR experiments were carried out using a Bruker E-500 spectrometer equipped with an ER 4122SHQE resonator and a Super-X FT Bridge. Samples of **1**, **2**, and **3** were prepared in a 2 mm capillary tube and placed in a quartz EPR tube (4 mm diameter, 250 mm long). A septum was placed on the EPR tube, and the sample was degassed using a vacuum line setup. A line needle was inserted into the septum of the EPR tube; the vacuum was turned on for 30 min, and the pressure dropped to approximately 0.5 mbar. Scans were performed at room temperature at 297 and 60 K. The EasySpin toolbox^44^ was utilized to fit the EPR spectra for all samples at both 297 and 60 K. The *g*-factors were determined via EasySpin fitting and calibration of the magnetic field against a DPPH standard. The scan parameters and fitting procedures are listed in the Supporting Information.

### 4.4 Three-Dimensional Electron Diffraction

Three-dimensional electron diffraction (3D ED) was used to analyze the solid particles of the three precipitates. A FEI Tecnai G2 20 microscope (200 kV, λ = 0.0251 Å) with an LaB_6_ cathode equipped with an ASI Cheetah direct-detection camera (24-bit) was employed. The samples were deposited on a Cu Holey-carbon TEM grid, which was inserted into the cooling holder and cooled to N_2_(*l*) temperature. The samples were then inserted into the microscope via cryo-transfer and cooled to 96 K (sample holder tip temperature). The holder was heated to 183 K due to large amounts of ice crystals present on the grid’s surface; diffraction pattern measurements of **1** and **2** were done at this temperature. For **3**, after the sublimation of ice crystals, the holder was cooled to 153 K to measure the diffraction pattern. Continuous-rotation 3D ED with a data frames semiangle of 0.15^◦^ was used to measure diffraction patterns, which were analyzed using the program PETS2.^9^ The structure of Pb_2_^4+^(C_6_O_6_)^2–^Cl_2_^2–^· 3H_2_O was solved by SHELXT^45^ and refined by Jana2020.^46^ Overlapping virtual frames with semiangle 1.35^◦^ were used.

### 4.5 Ancillary Techniques

FT-IR measurements were collected on the Thermo Fisher Scientific Nicolet iS50 FTIR system equipped with a Smart iTX with a monolithic ATR. PXRD patterns were collected on a Bruker D8 Discover General Area Detector Diffraction System (GADDS) equipped with a VÅNTEC-2000 2D detector and a Cu–Kα source (λ = 1.54178 Å) to analyze the crystallinity and structure of these compounds. A graphite crystal was used to monochromate the X-ray beam and collimated with a 0.5 mm capillary collimator (MONOCAP). Powders of the three compounds were deposited directly onto a silicon wafer, and diffraction patterns were collected in reflection mode. Powder samples, investigated 9 months after synthesis, were measured at room temperature (**1**) or at 150 K (**2**, **3**) in a 0.5 mm borosilicate glass capillary in a Debye–Scherrer transmission configuration using the PANalytical powder diffractometer Empyrean. UV–vis spectra were measured using a Qmini Miniature USB Spectrometer (λ = 380–750 nm). Due to the insolubility of the compounds, the precipitates were crushed onto lens paper and coated in castor oil to promote the transparency of the paper. This was successful for measuring spectra of **2** and **3**.

## 5 Conclusions

We have synthesized and analyzed the three precipitates (**1, 2, 3**) that are formed as part of the rhodizonate lead detection test using 3D ED and magnetic resonance. The structural transformations in the disordered and ordered components of the precipitates were followed, and it was found that the coordination of the lead site as well as the electron density about the rhodizonate changes significantly between **1**, **2**, and **3**. The rhodizonate test for Pb has been a widely used method of chemical analysis that is based on transformations among highly insoluble coordination polymers. The rise of 3D ED has enabled the structural characterization of one of the terminal nanocrystalline products of the spot tests. For those precipitates not even amenable to nanocrystal-structure determination, we supplemented their characterization with solid-state NMR and EPR data. Combining these approaches has allowed us, for the first time, to solve the structure of a lead rhodizonate complex and follow the local atomic-level structural changes associated with the lead rhodizonate spot test.

Interestingly, the color of all three samples remained similar 9 months after synthesis. The NMR and PXRD of **1, 2**, and **3** noticeably changed following this period. It is possible that the color changes observed in the sodium rhodizonate test are associated with structures having a high degree of disorder. A more thorough analysis of how the optical absorption evolves over time would be needed to further support this conclusion. From this study, it is clear that the precipitates formed as part of the sodium rhodizonate test consist of mixtures, and the chemistry of the components can evolve over long periods of time. In order to prevent chemical evolution of the precipitates, the results that are used in forensics should be evaluated within weeks of testing, and precipitates should be kept in a dry, and low-light environment. This work is a significant step toward an improved understanding of the factors that contribute to the optical properties of the forensic sodium rhodizonate test.

## Supplementary Material



## Notes

Additional details, including spin relaxation fits, figures, comparison spectra, and synthesis details, are described (PDF)
