# Supplementary material for: Structural Characterization of the Forensic Sodium Rhodizonate Test for Lead
Source: Inorg Chem. 2026 Sep 26;65(40):23424–32. doi: 10.1021/acs.inorgchem.6c02536 (PMC13664093; doi:10.1021/acs.inorgchem.6c02536)
Supplement: Supplementary file 1 [file ic6c02536_si_001.pdf]

Supporting Information

Structural Characterization of the Forensic Sodium Rhodizonate Test for Lead

Alex Kukucka,† Devin W. Thrush,† Trent A. McHenry,† Markéta Jarošová,‡ Talaidh Isaacs,† Anabelle Bahadur,† Jan Rohlíček,‡ Michelle Segura,† Alexander G. Shtukenberg,† Petr Brázda,‡ Bart Kahr,† and Claudia E. Avalos*,†

†*Department of Chemistry and Molecular Design Institute, New York University, New York City, New York 10003, United States*

‡*Department of Structure Analysis, Institute of Physics, Czech Academy of Sciences, 18200 Prague 8, Czech Republic*

E-mail: claudia.avalos@nyu.edu

Contents

1 Electron Diffraction (3D ED) S-3

2 Fourier Transform Infrared (FT-IR) Spectroscopy S-4

3 Powder X-Ray Diffraction S-5

4 Solid-State Nuclear Magnetic Resonance Spectroscopy S-12

4.1 ^{207}Pb NMR S-13

4.2 ^{1}H-^{13}C Cross Polarization (CP) S-14

4.3 ^{1}H NMR S-15

4.4 Sample Evolution S-18

4.5 Experimental Parameters S-19

5 Electron Paramagnetic Resonance **S-21**

5.1 Fitting S-26

1 Electron Diffraction (3D ED)

Table S1: Lattice parameters of components of product **2**.

Crystal name	a [Å]	b [Å]	c [Å]	γ[°]
C1	8.00	8.00	13.77	120
C2	7.98	7.98	13.70	120
C3	7.94	7.94	13.93	120
Average	7.97(3)	7.97(3)	13.80(9)	120

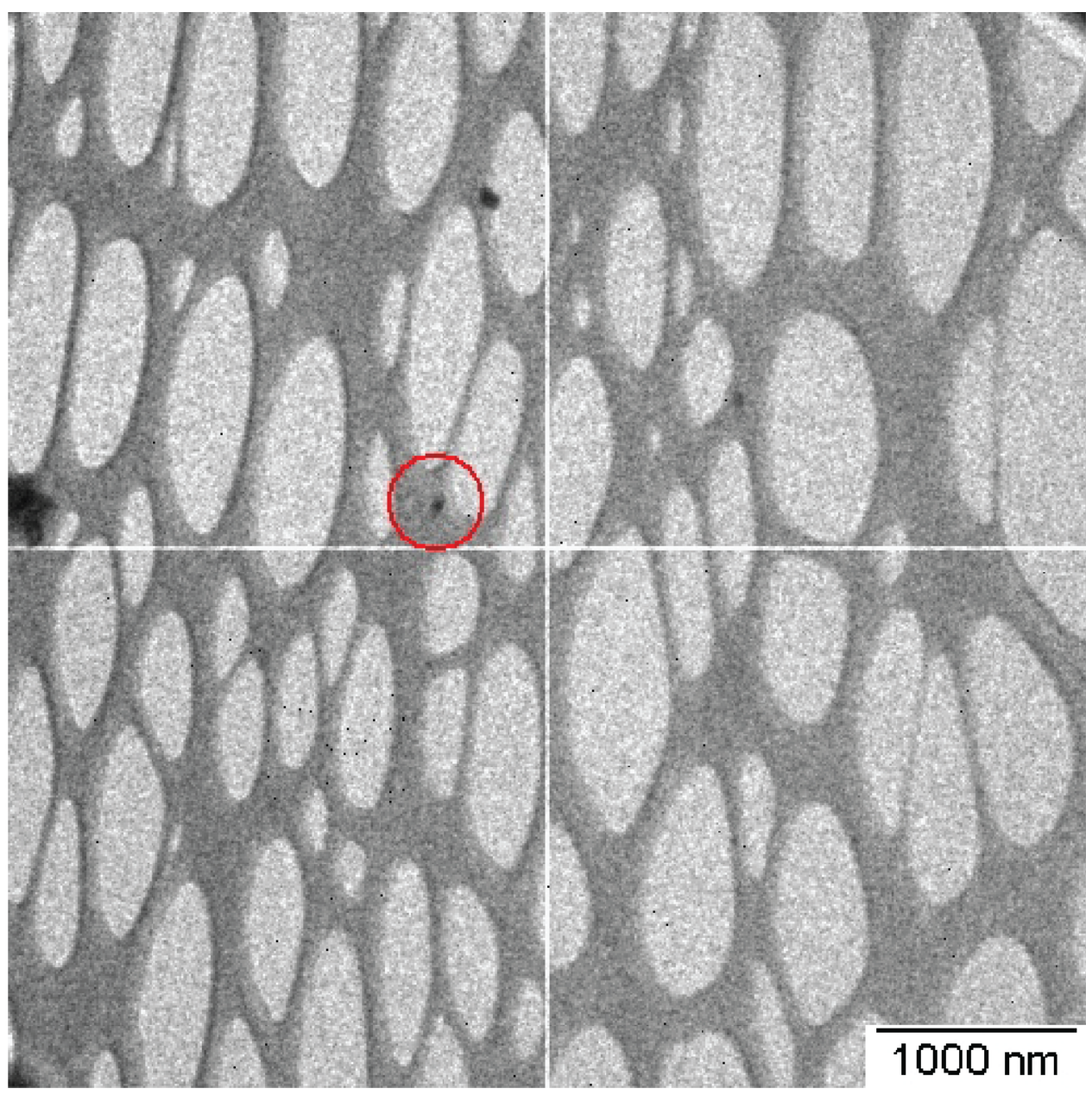

Figure S1: SEM Image of crystallites from precipitate **3** as prepared for 3D ED. Red circle marking one measured crystallite.

Table S2: Crystallites observed by 3D ED from **3**

# Datasets	Phase	a[Å]	b[Å]	c[Å]	α[°]	β[°]	γ[°]	Bravais class
26	$Pb_2^{4+}(C_6O_6)^{2-}Cl_2^{2-}\cdot 3H_2O$	8.07	8.07	16.57	90	90	120	hRobv
3	$PbCl_2$	7.59	4.51	9.02	90	90	90	oP
2	Talc-like	7.91	5.98	10.64	90	90	90	oP
2	K_2SO_4	5.44	5.44	9.56	90	90	120	hP
12	Impossible to index							

2 Fourier Transform Infrared (FT-IR) Spectroscopy

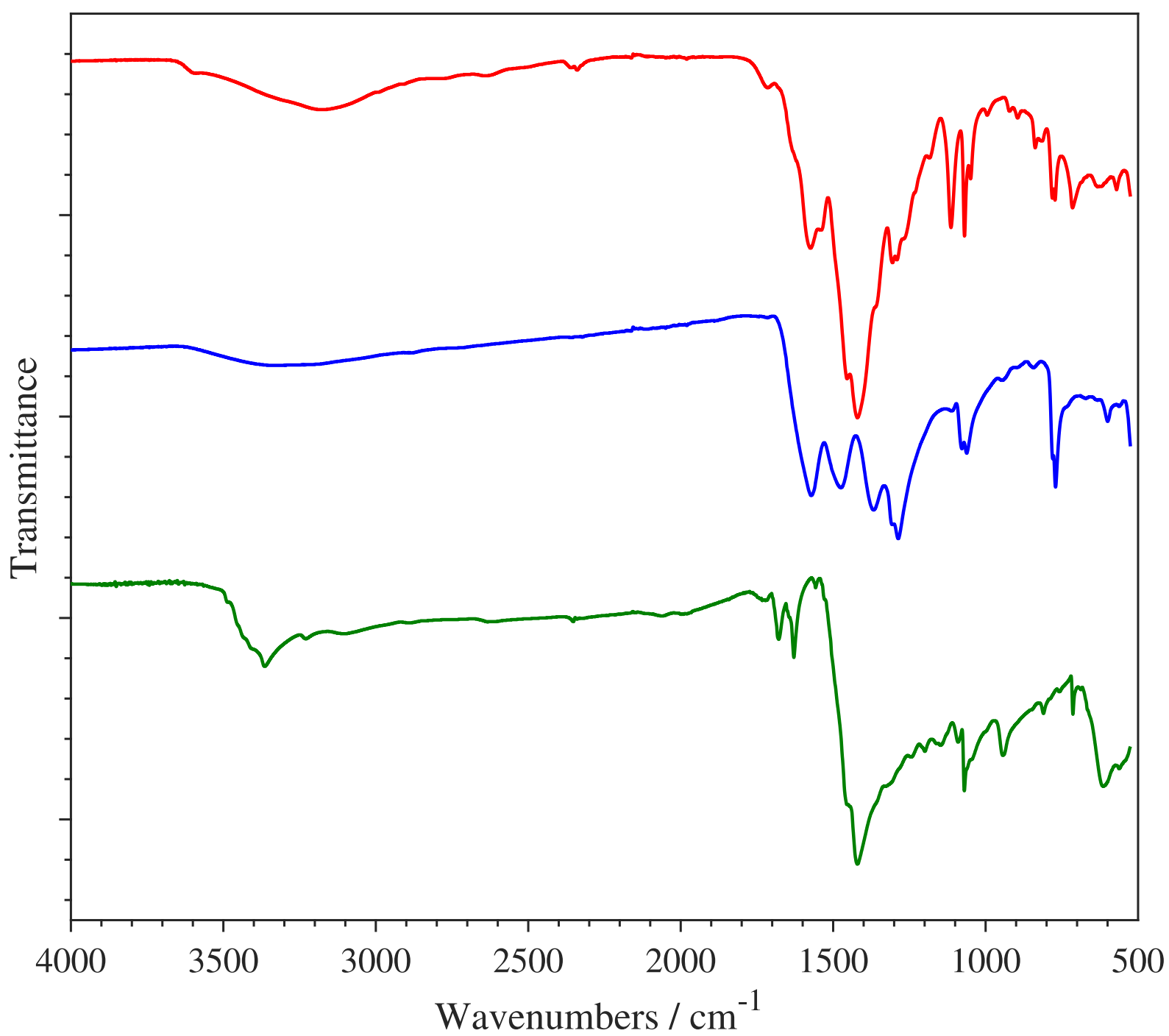

Figure S2: FT-IR spectra of **1** (dark blue), **2** (red), and **3** (green). See reference.[1]

3 Powder X-Ray Diffraction

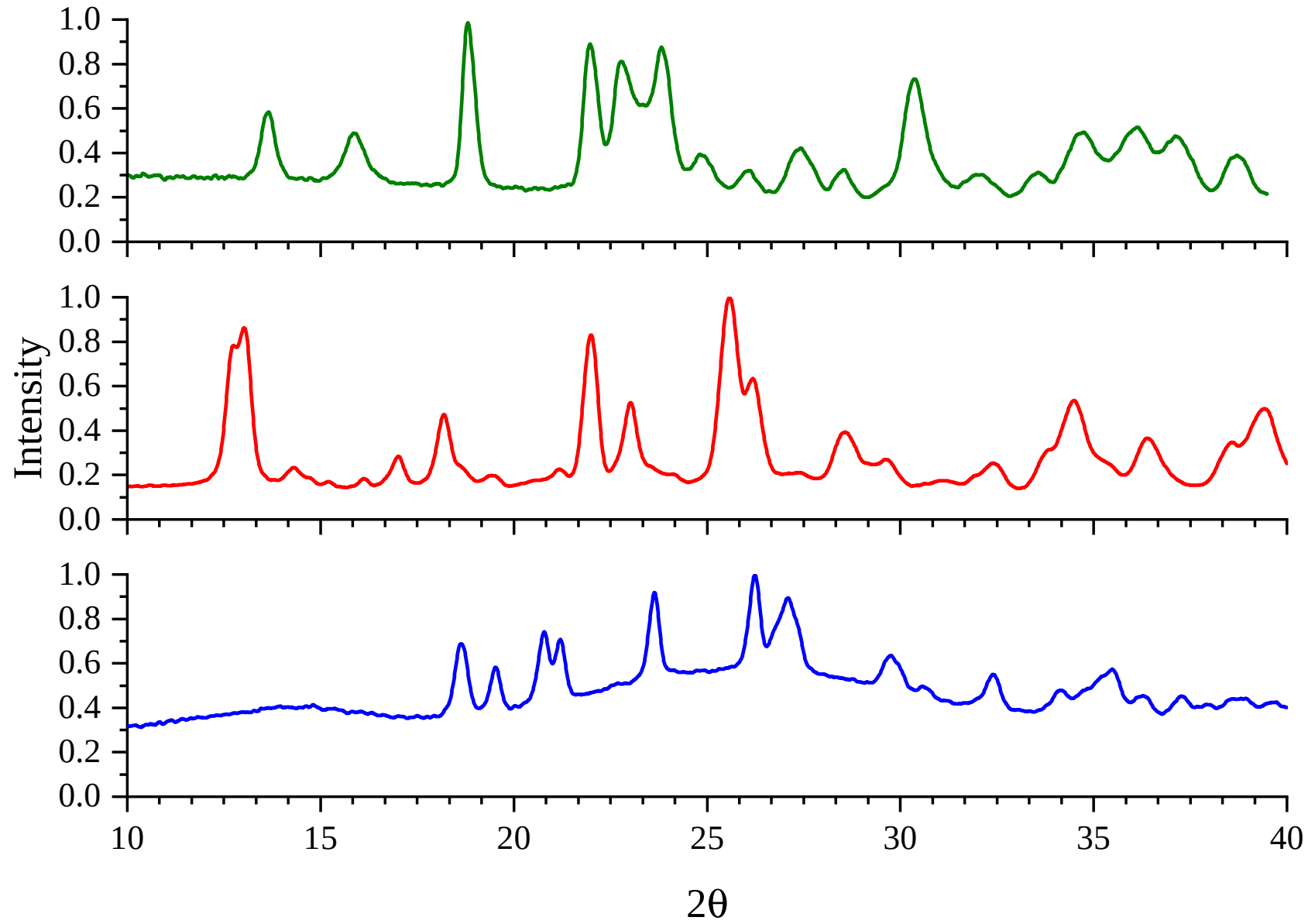

Figure S3: PXRD of **1** (blue), **2** (red) and **3** (green). Diffractograms normalized to highest individual intensity.

Clear differences between the diffraction patterns of **1**, **2**, and **3**, are observed (Figure S3). Powder diffraction (λ = 1.54178 Å) of **1**, shown in S3, revealed an amorphous halo. The diffraction pattern is distinct from that of the starting material sodium rhodizonate.[2] **2** exhibited a higher degree of crystallinity than **1** but was inadequate to solve the structure. **3** was the most crystalline, however, the resolution was still insufficient to solve the structure using PXRD alone, necessitating the implementation of 3D ED as described in the main text. Le Bail fits of PXRD data shown in Figure S3 are shown in the figures below.

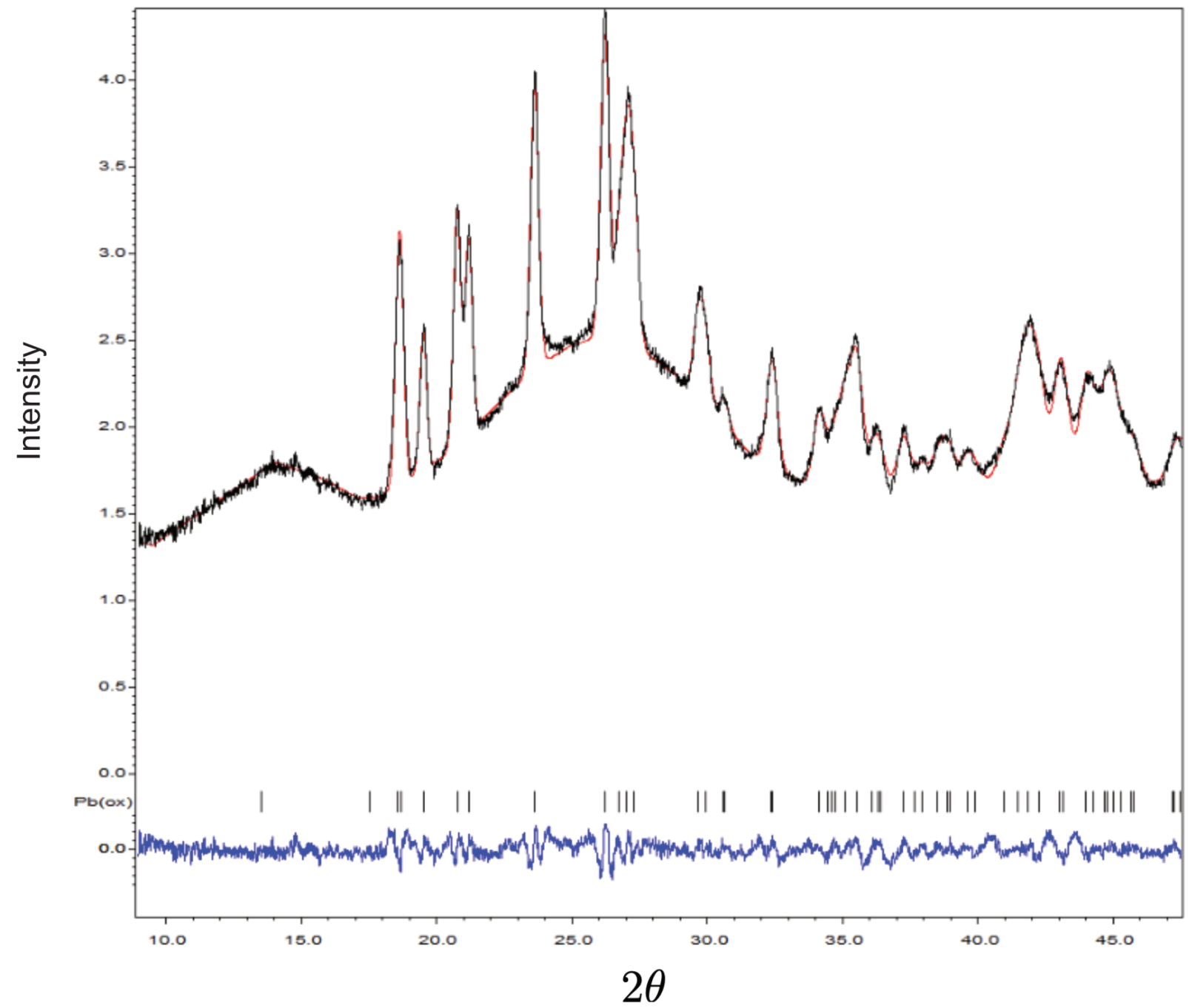

Figure S4: PXRD of **1**. Data - black, Le Bail fit - red, difference - blue, positions of lead oxalate diffraction maxima - black bars.

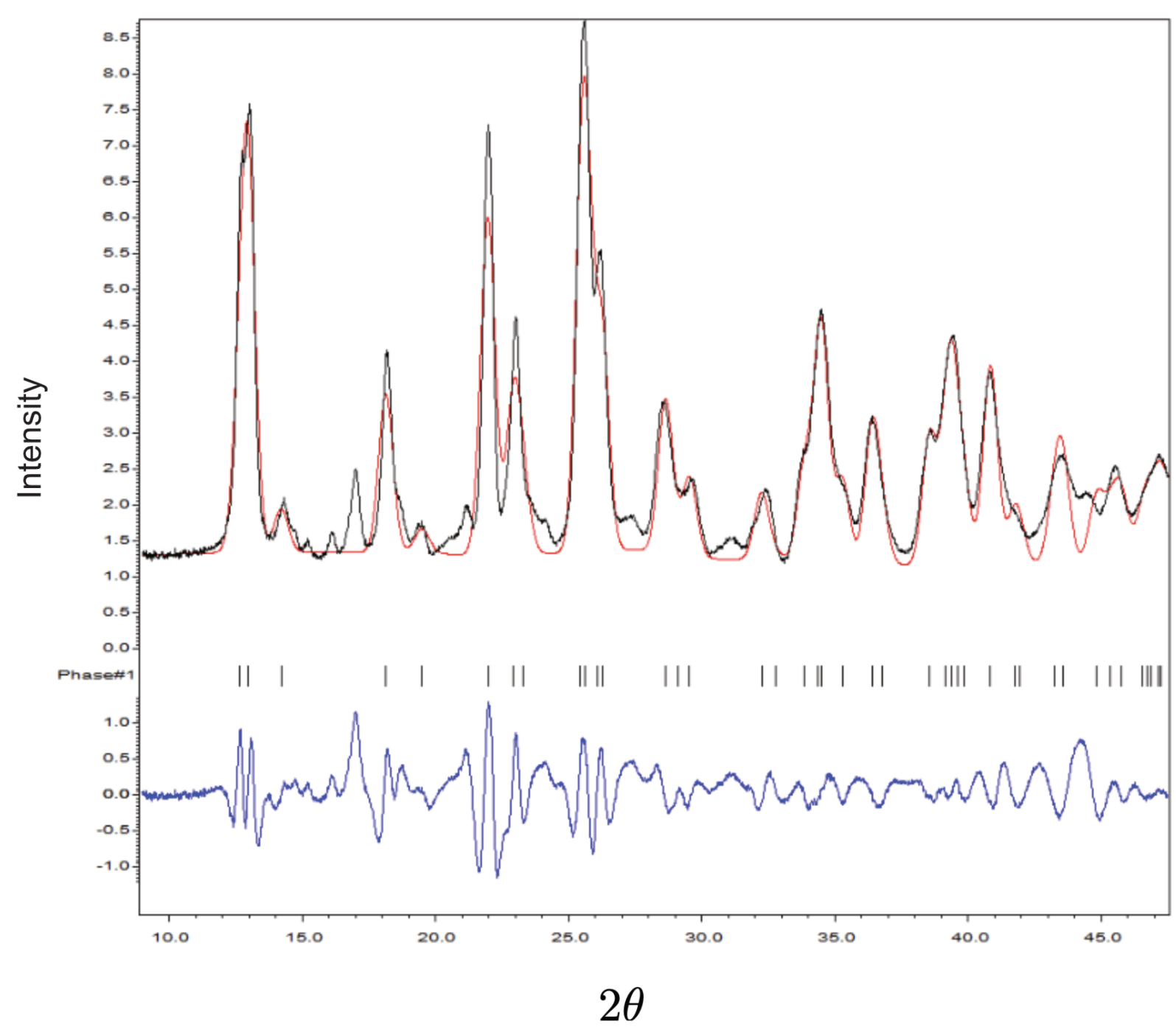

Figure S5: PXRD of **2**. Data - black, Le Bail fit - red, difference - blue, positions of **2** diffraction maxima - black bars.

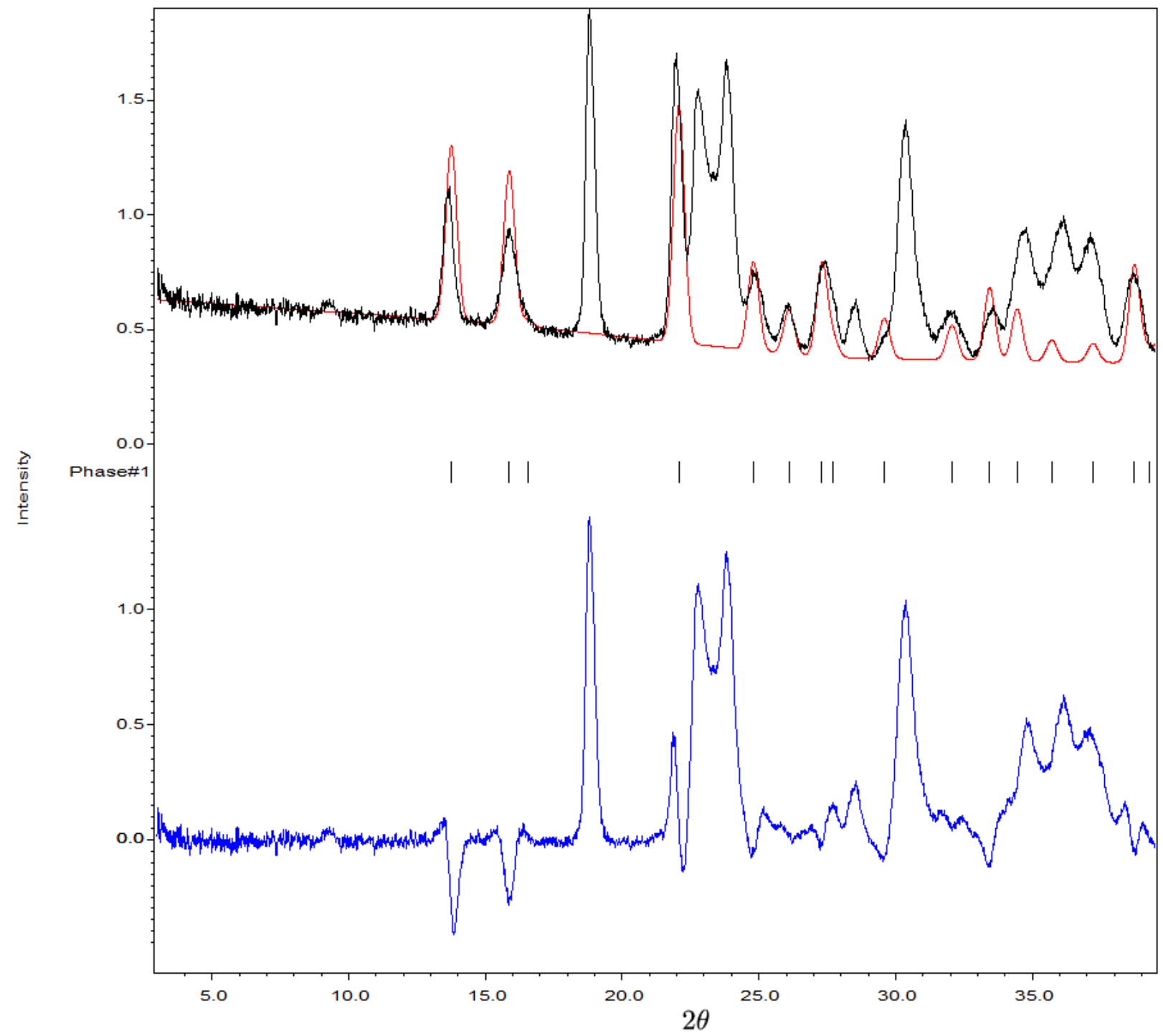

Figure S6: PXRD of **3**. Data (measured at 300 K) - black, fixed structure model from 3D ED (measured at 150 K) - red, difference - blue, positions of 3D ED solved **3** structure diffraction maxima - black bars.

A proper comparison of intensities between 3D ED and PXRD is not possible as the PXRD was taken at room temperature and the 3D ED at 150 K. Because of this, the displacement parameters will be different even if the lattice parameters do not differ substantially as shown in Figure S6. It can also be seen that above 35 deg there is a bad match between the PXRD data and 3DED model. This likely arises due to the presence of diffracted intensity from a secondary phase.

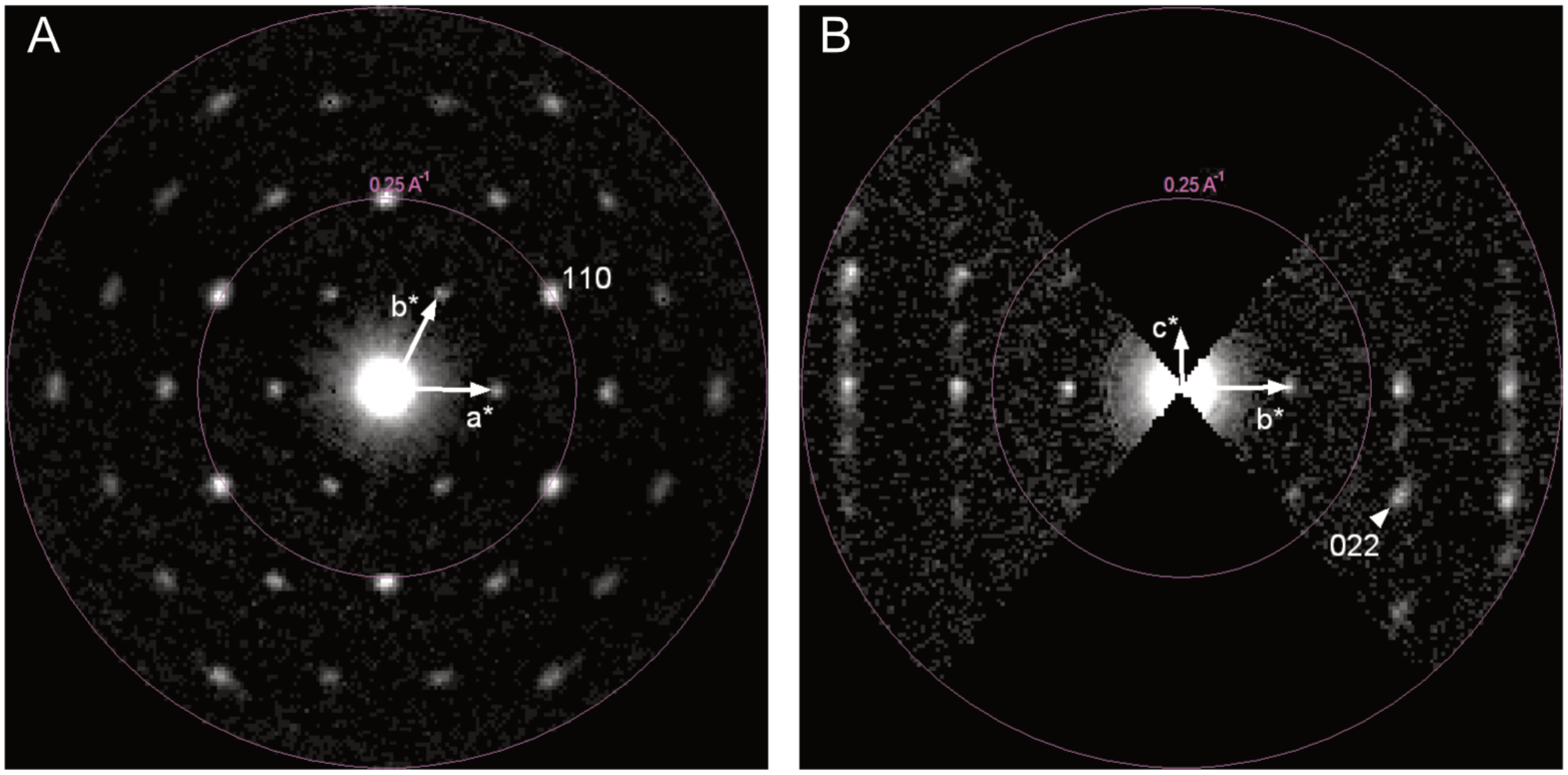

Figure S7: Reflections {110} of **2**. (A) $hk0$ reciprocal space section. (B) $0kl$ reciprocal space section.

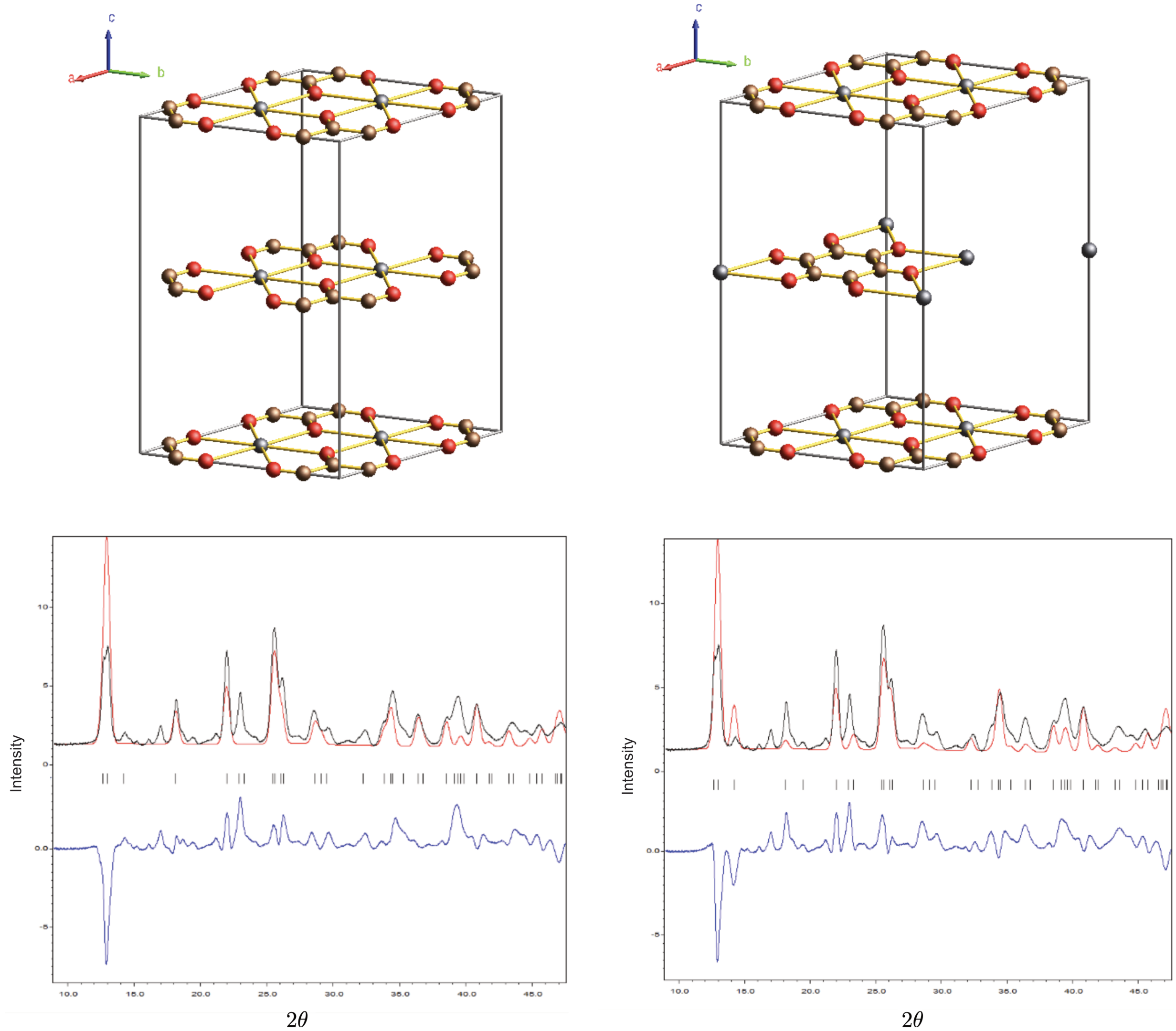

Figure S8: Structural models and Rietveld refinements against PXRD of **2**. (Left) Model 1, (Right) Model 2. (Bottom row) Rietveld refinements. Data - black, Rietveld fit - red, difference - blue, positions of diffraction maxima - black bars.

PXRD was measured on all three samples following a prolonged period (∼ nine months after synthesis. Lead oxalate is one of the decomposition products in these materials. These results are shown below.

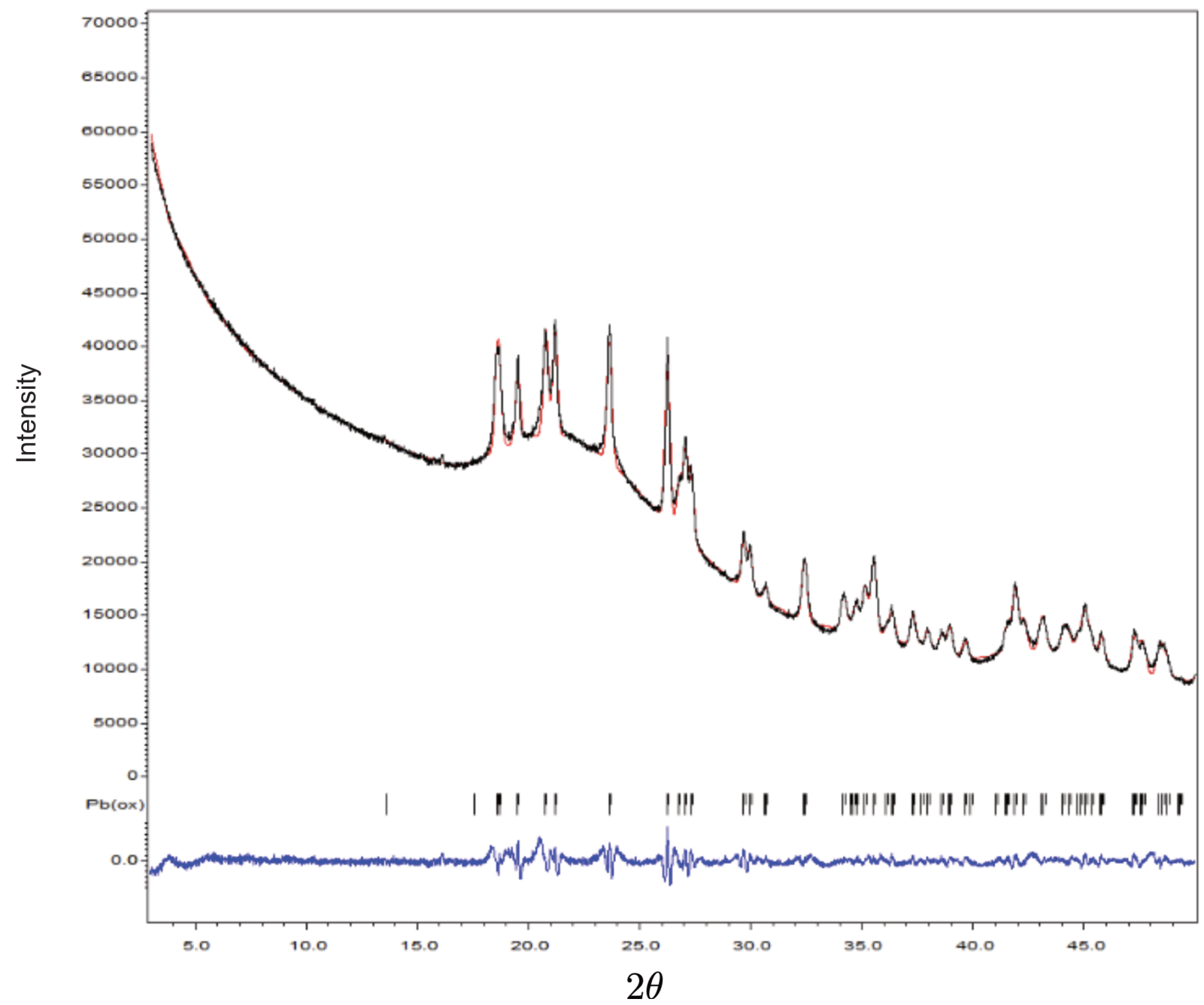

Figure S9: PXRD of **1**. Data - black, Le Bail fit - red, difference - blue, positions of lead oxalate diffraction maxima - black bars.

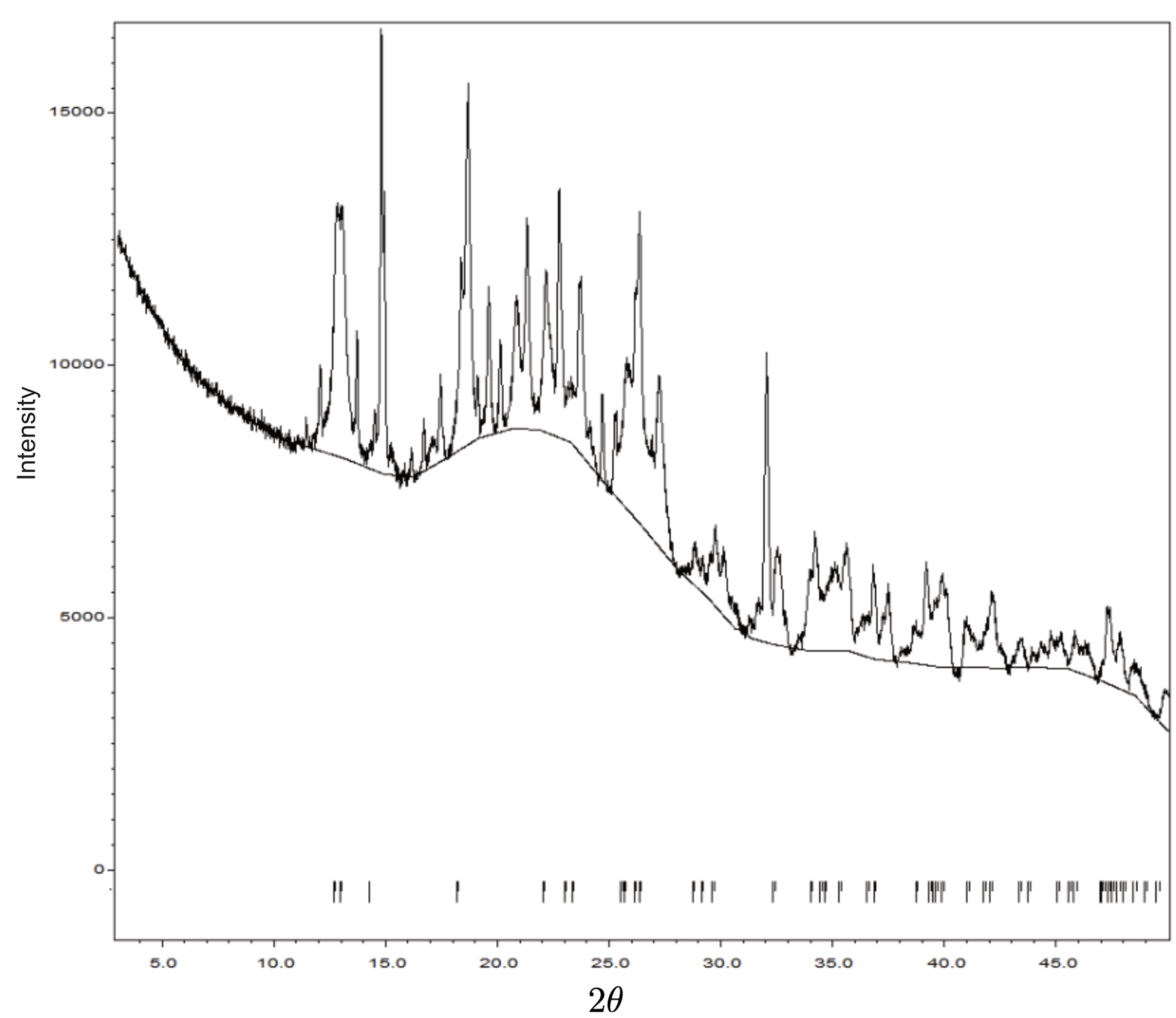

Figure S10: PXRD of **2**. Data - black, positions of **2** diffraction maxima - black bars.

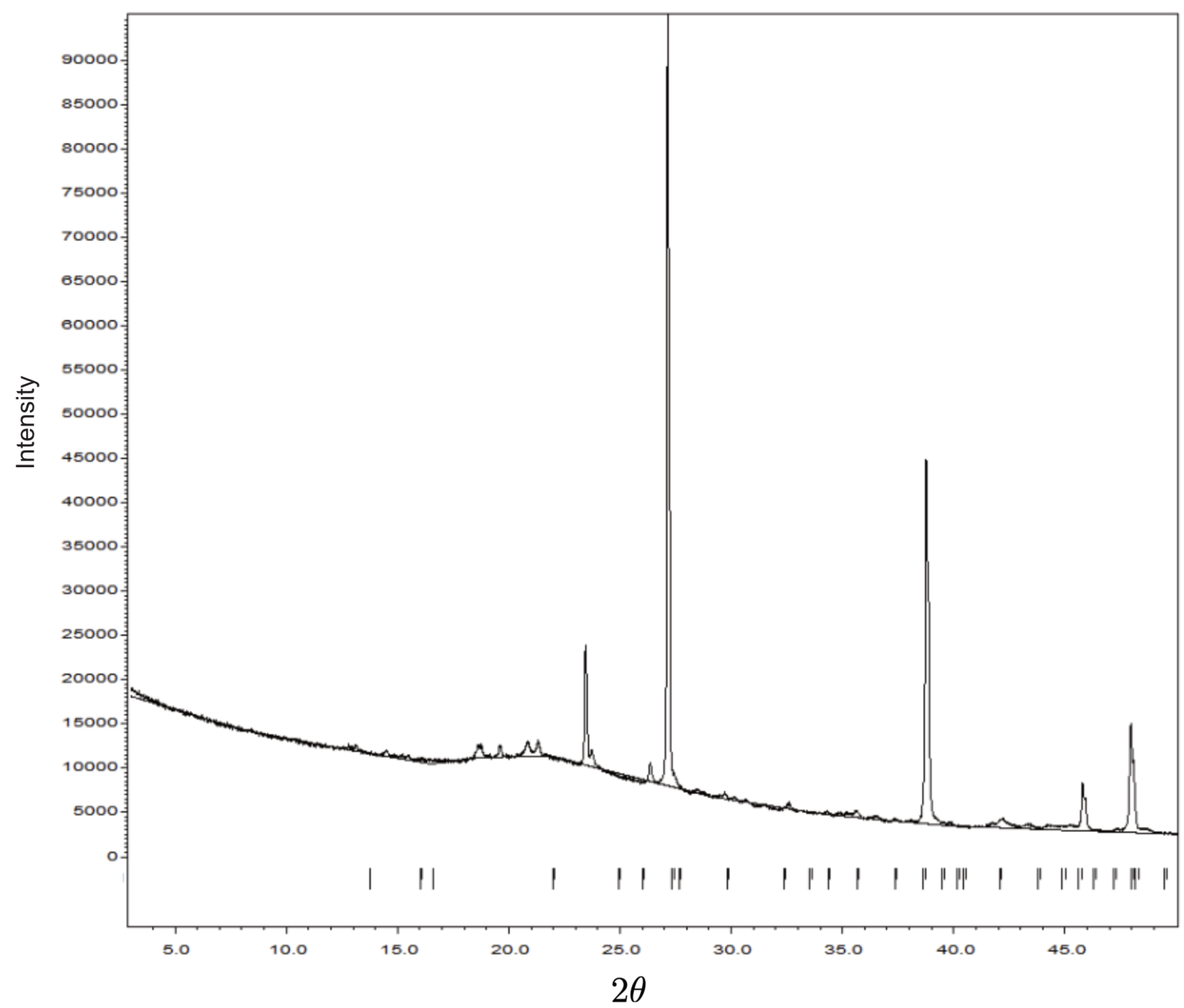

Figure S11: PXRD of **3**. Data - black, positions of 3D ED solved **3** structure diffraction maxima - black bars.

4 Solid-State Nuclear Magnetic Resonance Spectroscopy

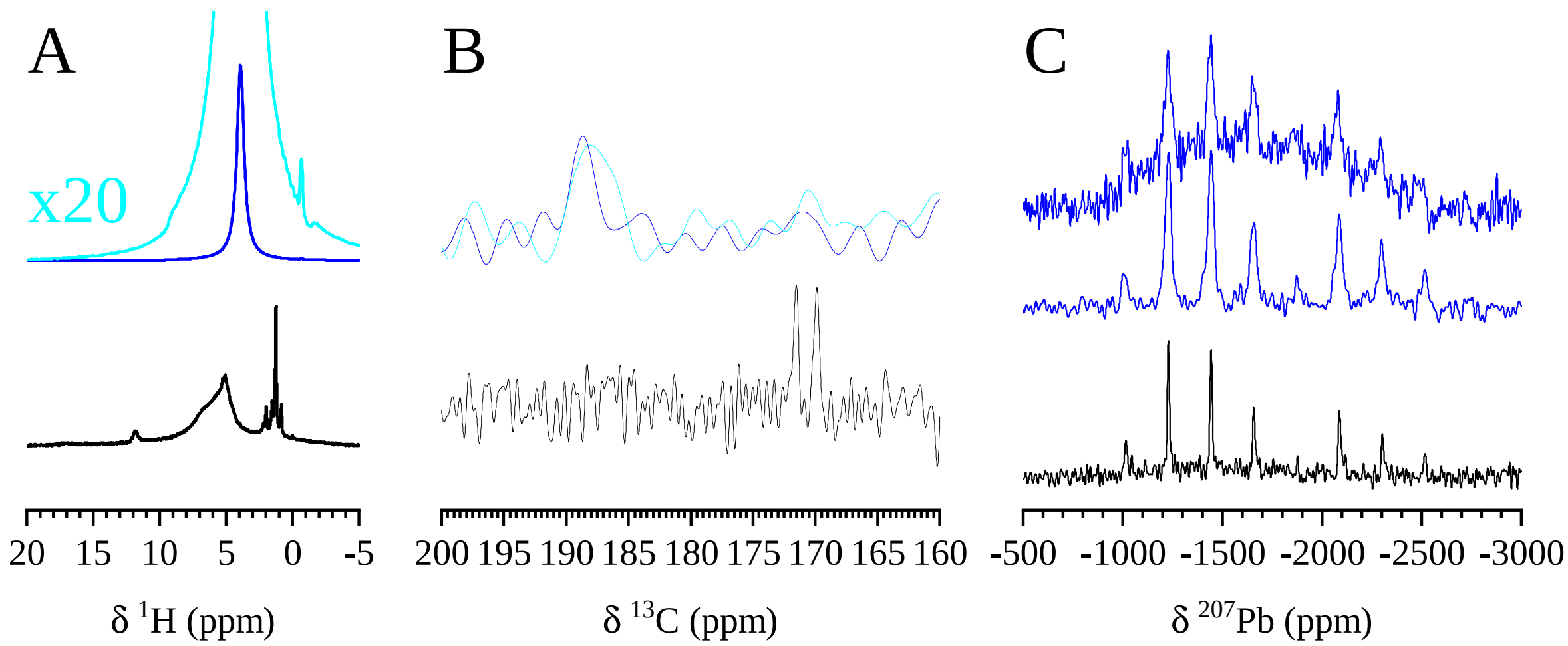

Figure S12: (A) 1D proton NMR (^{1}H) of **1** (blue and cyan) and lead oxalate (black). (B) 1D carbon NMR (^{13}C) of **1** (blue and cyan) and lead oxalate (black). **1** presents a single feature at 188.7 ppm, while lead oxalate displays 2 peaks; 171.5 and 169.8 ppm. (C) 1D lead NMR (^{207}Pb) of **1** (blue, top: short echo, bottom: long echo) and lead oxalate (black). Fitting parameters for lead spectra are given in table S3. Experimental parameters are given in table S3.

If we compare the lead NMR of **1** to that of lead oxalate as well as the fits obtained in Table S3 the parameters are nearly identical, consistent with the PXRD results showing the presence of lead oxalate in **1**. If we compare the carbon NMR of **1** to that of lead oxalate there are some notable differences. We observe a broader signal in **1** at a chemical shift of 188.7 ppm and a much narrower signal in lead oxalate at 171.5 and 169.8 ppm. The lead oxalate provides a small contribution to the overall signal in **1** and may be lost in the noise (Figure S12B). Comparing the ^{1}H NMR, Figure S12A, peaks associated with lead oxalate in **1** are obscured by the ^{1}H resonance associated with water.

Table S3: δ^{207}Pb fitting parameters for **1** and lead oxalate

Fitting parameters	δ(iso) (ppm)	Ω	κ
**1** (long echo delay)	-1658.0	1596.0	1.0
Lead Oxalate	-1658.2	1649.0	1.0

Table S4: Acquisition parameters of spectra shown in Figure S12

parameter	**1** (^{1}H)	lead oxalate (^{1}H)	**1** (^{13}C) (blue)	**1** (^{13}C) (cyan)	lead oxalate (^{13}C)	**1** (^{207}Pb)	lead oxalate (^{207}Pb)
Recycle Delay (s)	5	0.5	13	80	80	16	26.5
Dwell Time (μs)	6.1	5	10	10	10	1	1
MAS (kHz)	18	18	18	18	18	18	18
$\pi/2$ pulse (μs)	4.6	4.6	3.5	1.95	1.95		
NS	16	512	256	128	256	2048	1024
Transmitter Offset (ppm)	0	1.55	100	100	100	-1700	-1700
^{1}H Decoupling Program	-	-	Spinal64	Spinal64	Spinal64	-	-
^{1}H Decoupling amplitude	-	-	109 kHz	109 kHz	109 kHz	-	-

4.1 ^{207}Pb NMR

Saturation recovery curves were fit to a monoexponential using the T1T2 module of Topspin using the following equation $y = A * (1 - exp(\frac{-x}{t})) + b$ (A: amplitude, t: time constant, b: y intercept). T_1 of **1**, **2**, **3** was measured to be 7.0 ± 0.9 s, 39 ± 3 ms, and 3.2 ± 0.4 s for **3**. The quality of the datasets for **2** and **3** were poor in part due to the low signal to noise obtained for these samples.

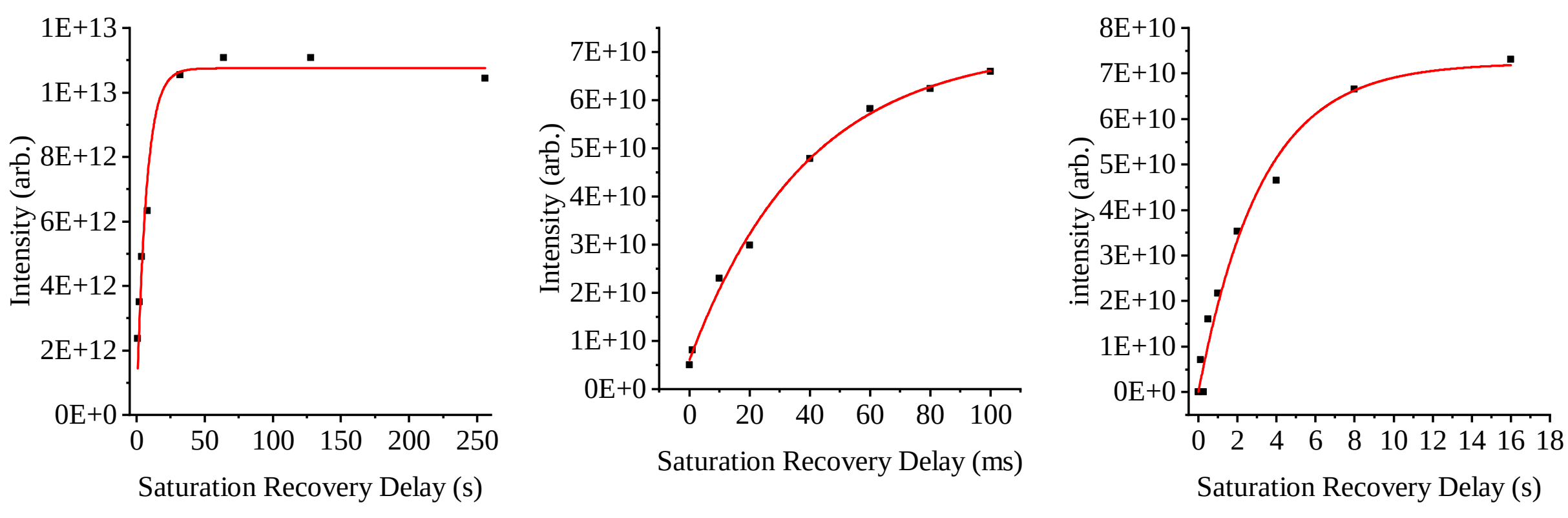

Figure S13: Saturation recovery ^{207}Pb NMR at 275 K, 18 kHz. **1** (left), **2** (center) and **3** Integrated areas are plotted against recycle delay with monoexponential fits shown.

4.2 ^{1}H-^{13}C Cross Polarization (CP)

^{13}C signal via CP was detected in **2, 3**, but not in **1**. The absence of signal in **1** is likely due to the absence of protons in close proximity to the rhodizonate ring rather than mobility effects. ^{1}H NMR measurements shown in the main text show similar linewidths in **1** and **3**.

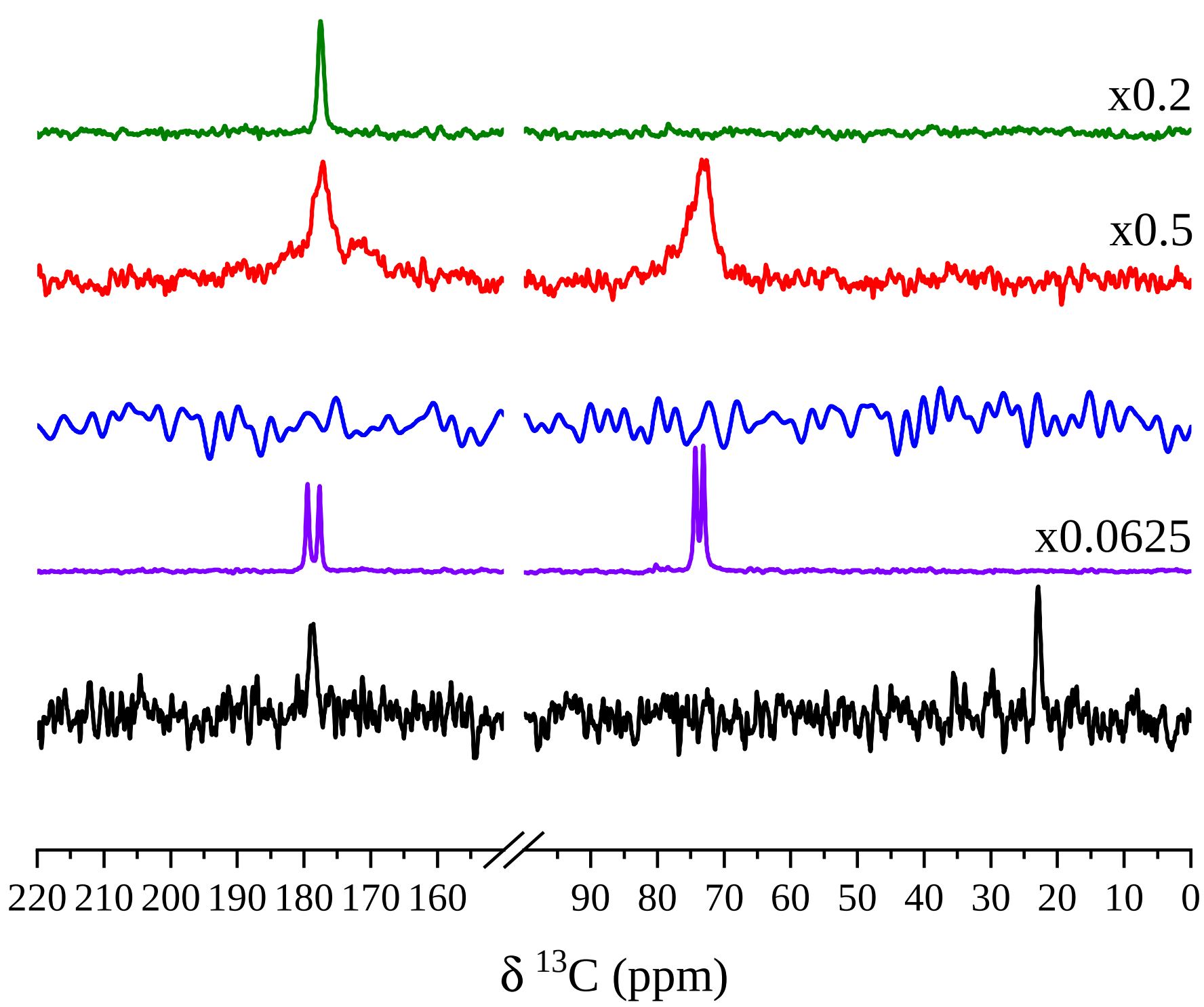

Figure S14: ^{13}C solid-state NMR CPMAS spectra at 10 kHz of starting material sodium rhodizonate (black), tartaric acid (violet), **1** (blue), **2** (red), **3** (green).

4.3 ^{1}H NMR

Isotropic chemical shifts of **1**, **2** and **3** are shown in table S5. The ^{1}H NMR spectra of tartaric acid and **2** are compared in Figure S15. ^{1}H T_1 measurements are summarized in Figure S16 and Table S6. A stretched exponential model was used to account for a distribution of T_1 values at each site. **1** exhibited the shortest T_1 of 262 ± 2 ms for the peak at 4.5(1) ppm along with a narrow T_1 distribution ($\beta = 0.974 \pm 0.008$). **2** exhibited longer average T_1 values of 766 ms ± 9 ms and $\beta = 0.95 \pm 0.01$. The average ^{1}H T_1 of **3**, 308 ± 5 ms, is similar to that of **1** and exhibits the broadest distribution of T_1 values $\beta = 0.82 \pm 0.02$.

Table S5: ^{1}H NMR: Isotropic chemical shifts δ_{iso} and Lorentizan linewidth fit parameters, Γ, as calculated using the multipeak tool and Levenberg-Marquardt method in OriginPro 2021. Reported errors are from the analytical fit.

Precipitate	δ_{iso} (ppm)	Γ (ppm)
**1**	4.5 ± 0.1	0.6518 ± 0.0008
**2**	5.1 ± 0.1<br>8.9 ± 0.1<br>14.5 ± 0.2	4.80 ± 0.03<br>3.4 ± 0.1<br>9.8 ± 0.8
**3**	2.2 ± 0.1<br>3.7 ± 0.1	0.953 ± 0.006<br>1.06 ± 0.03

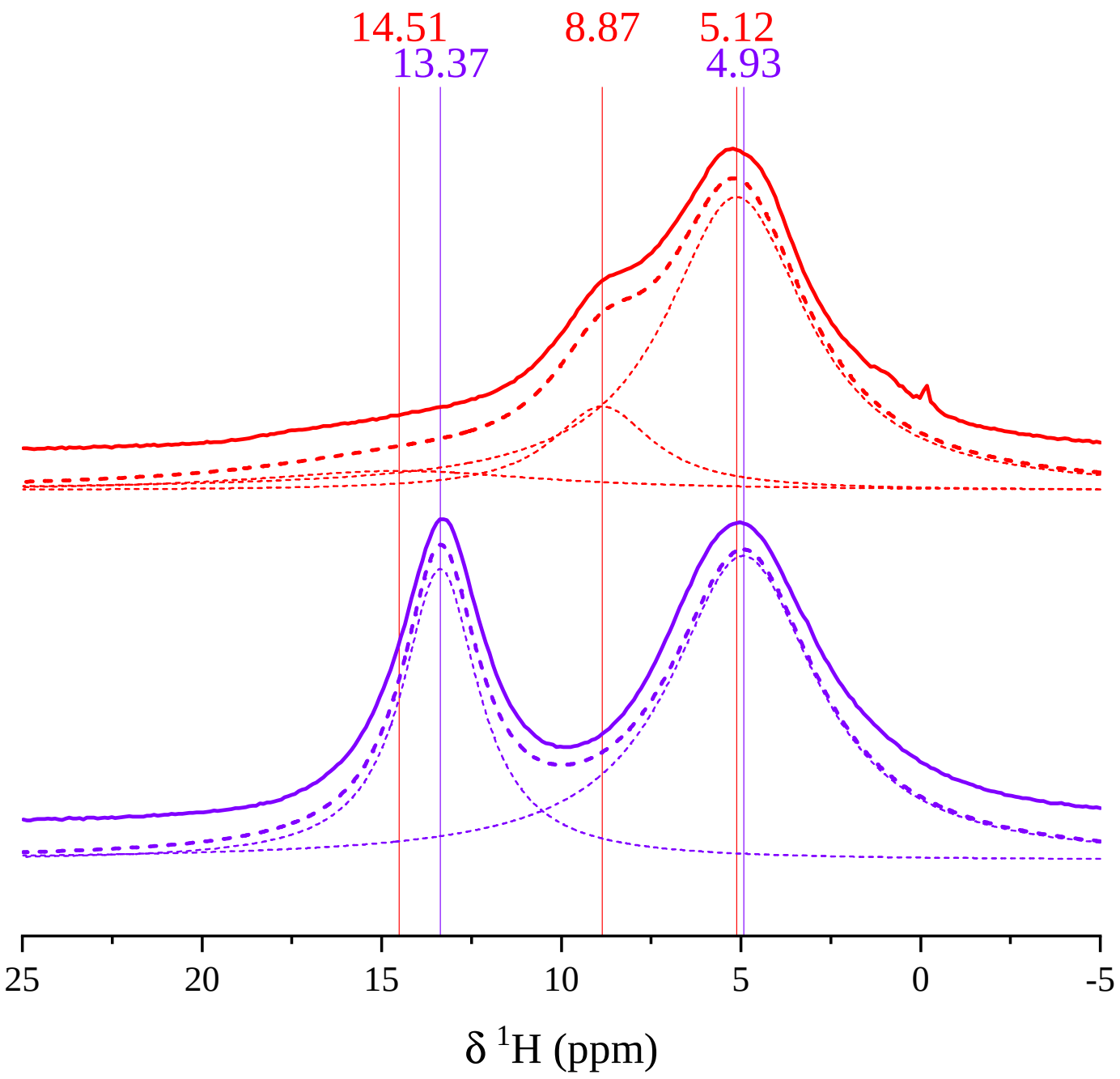

Figure S15: Comparison of ^{1}H spectra of tartaric acid (purple) and **2** (red).

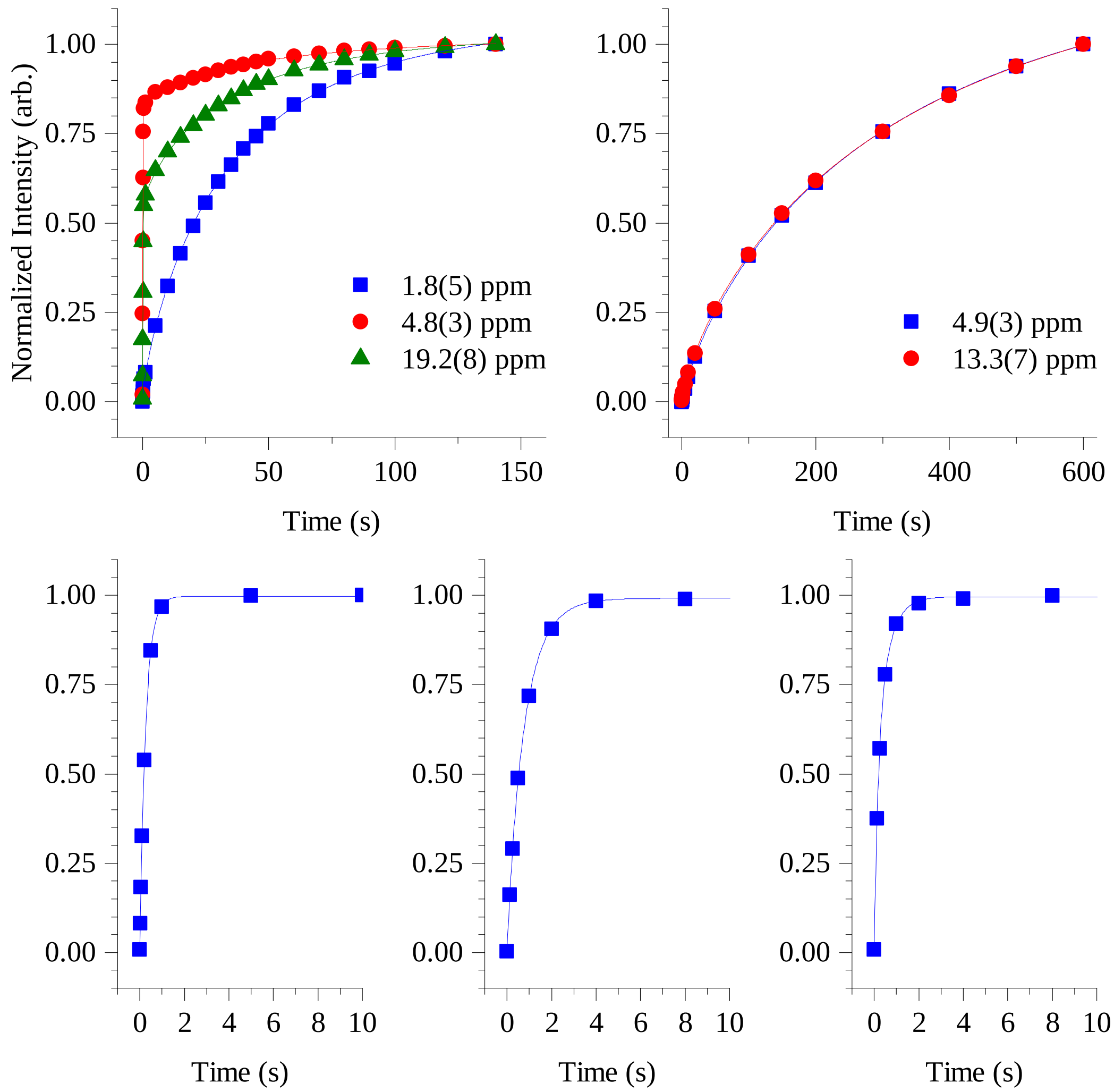

Figure S16: ^{1}H T_1 saturation recovery plots with stretched exponential fits (Equation S1, fitting results shown in table S6). Spectra were collected at 275 K at 18 kHz MAS. (a) Starting material sodium rhodizonate, 19.2(8) ppm (blue squares), 4.8(3) ppm (red circles), 1.8(5) ppm (green triangles). (b) tartaric acid, 4.9(3) ppm (blue squares), 13.3(7) ppm (red circles). (c) **1**, 4.5(1) ppm. (d) **2** and (d) **3**, were integrated across entire spectrum

Stretched exponentials may be used to describe NMR signals that contain a distribution of T_1 values.[3]

$$I(t) = A * (1 - e^{-(\frac{t}{T_1})^{\beta}}) + I_0 \tag{S1}$$

A is the amplitude, T_1 the relaxation time, β indicates the distribution of T_1 times (with $\beta = 1$ being a single site exponential recovery) and I_0 is the offset.

Table S6: T_1 and β parameters of saturation recovery fits.

Sample	δ_{iso}	Integration Region	T_1	β
Sodium Rhodizonate	1.9 ppm	1.675 : 2.274 ppm	126 ± 3 ms<br>35 ± 1 s	1.00 ± 0.03<br>0.78 ± 0.03
	4.8 ppm	5.269 : 4.571 ppm	67 ± 1 ms<br>42 ± 5 s	0.86 ± 0.02<br>0.810 ± 0.007
Tartaric acid	4.9 ± 0.1 ppm	4.239 : 6.007 ppm	320 ± 10 s	0.790 ± 0.009
	13.4 ± 0.1 ppm	12.739 : 14.013 ppm	337 ± 7 s	0.771 ± 0.005
**1**	4.5 ± 0.1	-3.197 : 13.157 ppm	262 ± 2 ms	0.974 ± 0.008
**2**	All Features	-13.887 : 24.549 ppm	766 ± 9 ms	0.95 ± 0.01
**3**	Both Features	-2.828 : 8.101 ppm	307 ± 5 ms	0.82 ± 0.02

4.4 Sample Evolution

^{1}H, ^{13}C and ^{207}Pb NMR Spectra were collected for all samples following a prolonged period of storage at ambient conditions for ∼ 9 months in order to compare the samples to a known lead oxalate sample. Comparisons are shown in figure S17. **1, 2** show some oxalate formation.

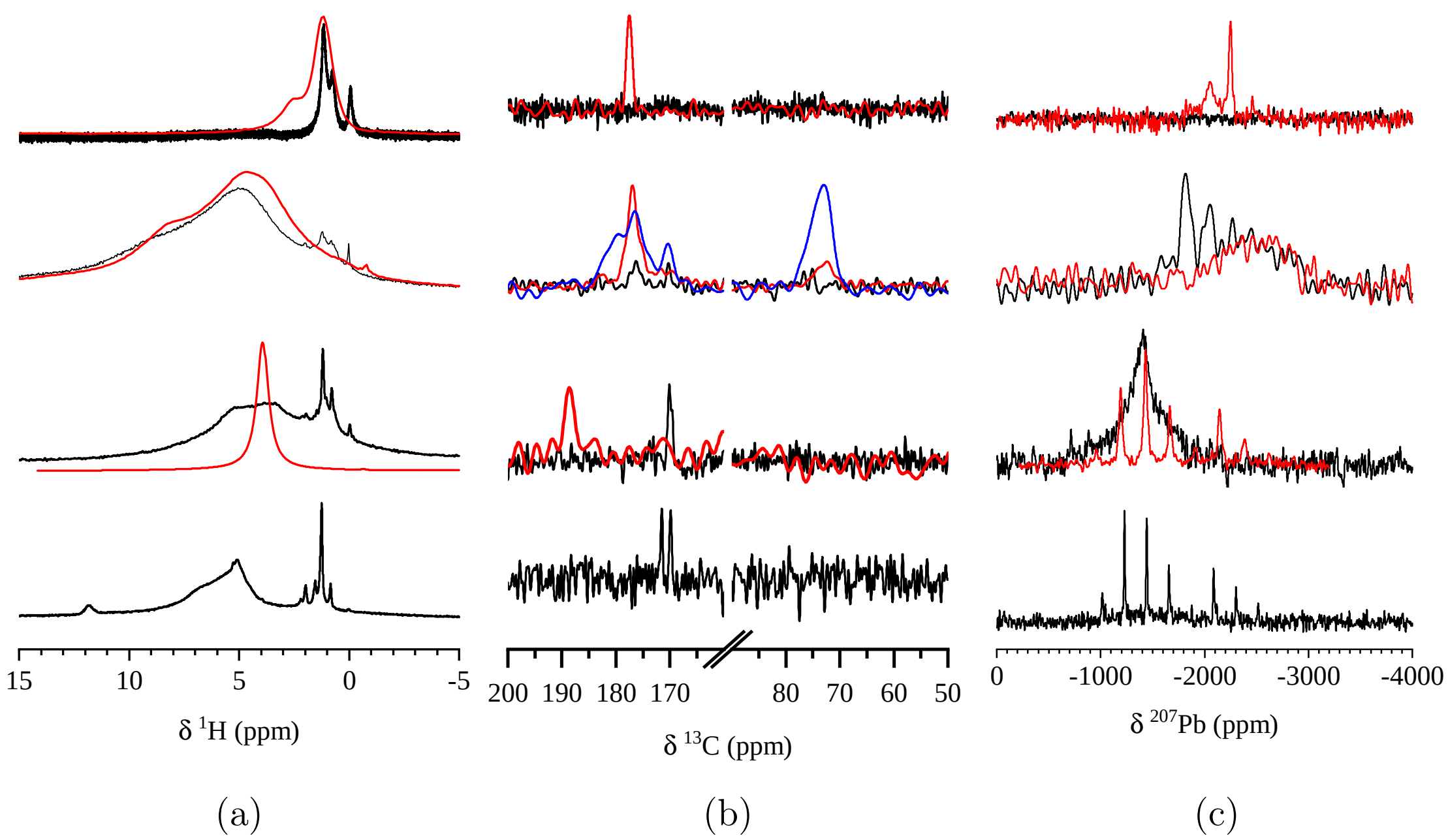

Figure S17: (a) ^{1}H NMR spectra, (b) ^{13}C NMR spectra and (c) ^{207}Pb NMR spectra of (bottom to top) lead oxalate (PbC_2O_4), **1**, **2**, and **3**. Red spectra of precipitates are as reported in main text. Black spectra are collected 9 months later. In the case of the carbon spectra of **2**, cross polarization (Blue), is also reported.

4.5 Experimental Parameters

Sidebands were identified by repeating measurements at multiple MAS spin rates. 109 kHz of decoupling power was used during ^{13}C NMR acquisition.

Table S7: Experimental parameters ^{207}Pb NMR echo ($\pi/2$-τ-π) spectra

Parameter	**1**	**2**	**3**
Recycle Delay (s)	16	0.4	16
Dwell Time (µs)	1	1	1
MAS (kHz)	18	18	18
$\pi/2$ pulse (µs)	1.95	1.95	1.95
NS	2048	131072	2048
Transmitter offset (ppm)	-1700	-2500	-2500

Table S8: Experimental Parameters for ^{13}C, ^{1}H 1D NMR echo measurements for all samples

Parameter	^{13}C	^{1}H
Relaxation Delay	10 s	1 s
Dwell Time	16.6 µs	3.1 µs
MAS	18 kHz	18 kHz
$\pi/2$ pulse	3 µs	2.75 µs
NS	256	16 (sodium rhodizonate,**1**)<br>4(tartaric acid,**2**,**3**)
Echo delay	55 µs	55 µs
^{1}H Decoupling Program	Spinal64	-
^{1}H Decoupling amplitude	109 kHz	-

5 Electron Paramagnetic Resonance

CWEPR measurements at 60 K (Figure S18) and at room temperature (Figures S19,S20) are shown below. EPR spectra of sodium rhodizonate, **1**, **2**, and **3** are shown in Figures S21 through S24. Fits are shown in black, and fitting parameters are provided in Table S9. The additional sharp feature present in the middle of the EPR spectrum of **1** does not relate to hyperfine or g-anisotropy of either of the two simulated radicals and its origin has not been identified, although its resonance appears in a region typical of oxocarbon radicals.

It was not possible to accurately fit ^{13}C hyperfine components on **1**, **2**, and **3** due to poor resolution and contributes to the margin of error for variation of g_{iso} values between fits. ^{13}C hyperfine was only resolved for the sodium rhodizonate case. Hyperfine values of A_1= 11.02 MHz, A_2= 5.29 MHz, and A_3= 17.61 MHz were obtained. These values are lower than literature values of rhodizonate radicals stabilized by MgO surfaces, consistent with a decreased unpaired electron spin density on carbon in the case of sodium rhodizonate.[4,5]

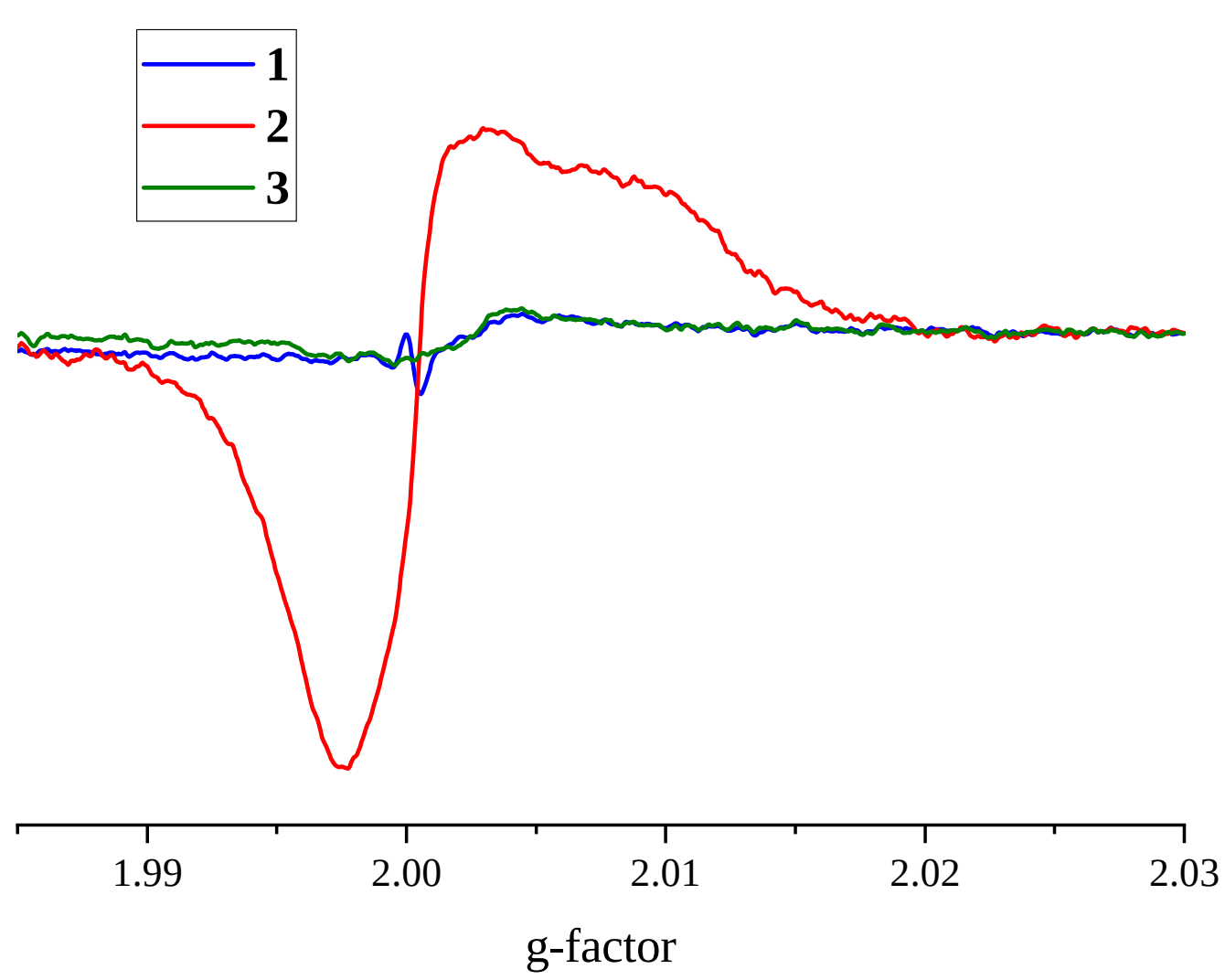

Figure S18: cwEPR of **1** (blue), **2** (red),**3** (green) at 60 K, plotted by g-factor

Table S9: 60 K Fitting Parameters at 0.0015 mW, the (') indicating a second component.

Chemical Species	g_1	g_2	g_3	g_{iso}	Relative Contribution
Sodium rhodizonate	2.0053	2.0051	2.0036	2.0046	
**1**	2.0033	2.0017	1.9861	1.9970	23.2%
**1'**	2.0048			2.0048	76.8%
**2**	2.0099	2.0006	1.9972	2.0025	
**3**	2.0035	2.0032	2.0005	2.0024	40.2%
**3'**	2.0053			2.0053	59.8%

Wider EPR scans were initially conducted to identify signals of interest. The results of these are shown in Figure S25. The organic signal near 350 mT was chosen for focus of study due to it being present across all samples. The additional signal present in **2** has not been assigned, although we suspect it is associated with an iron contaminant.

We find that g_{iso} for **1**, **2** and **3** are all lower than the g_{iso} of the organic radical in the starting materials sodium rhodizonate (Table S9, Figure S20). Bidentate binding of lead to oxygen substituents in the stable radical gallic acid leads to a lower g-factor relative to the unbound form.[6] Upon binding, radical density increases on the carbons within the ring.[6] A decrease in the radical density on the p-orbitals of the oxygen would lead to a decrease in the spin-orbit coupling contribution to the g-factor and a lower g-factor.[7] This suggests that for rhodizonate centered radical species within our samples, the radical density on carbon would be the highest for **1**, followed by **2** and **3**, and lastly sodium rhodizonate. The g-factor is also impacted by differences in water solvation. Water solvation affects the mixing between the ground and excited states through stabilization of non-bonding orbitals via hydrogen bonding, leading to a g-factor closer to that of a free electron.[8]

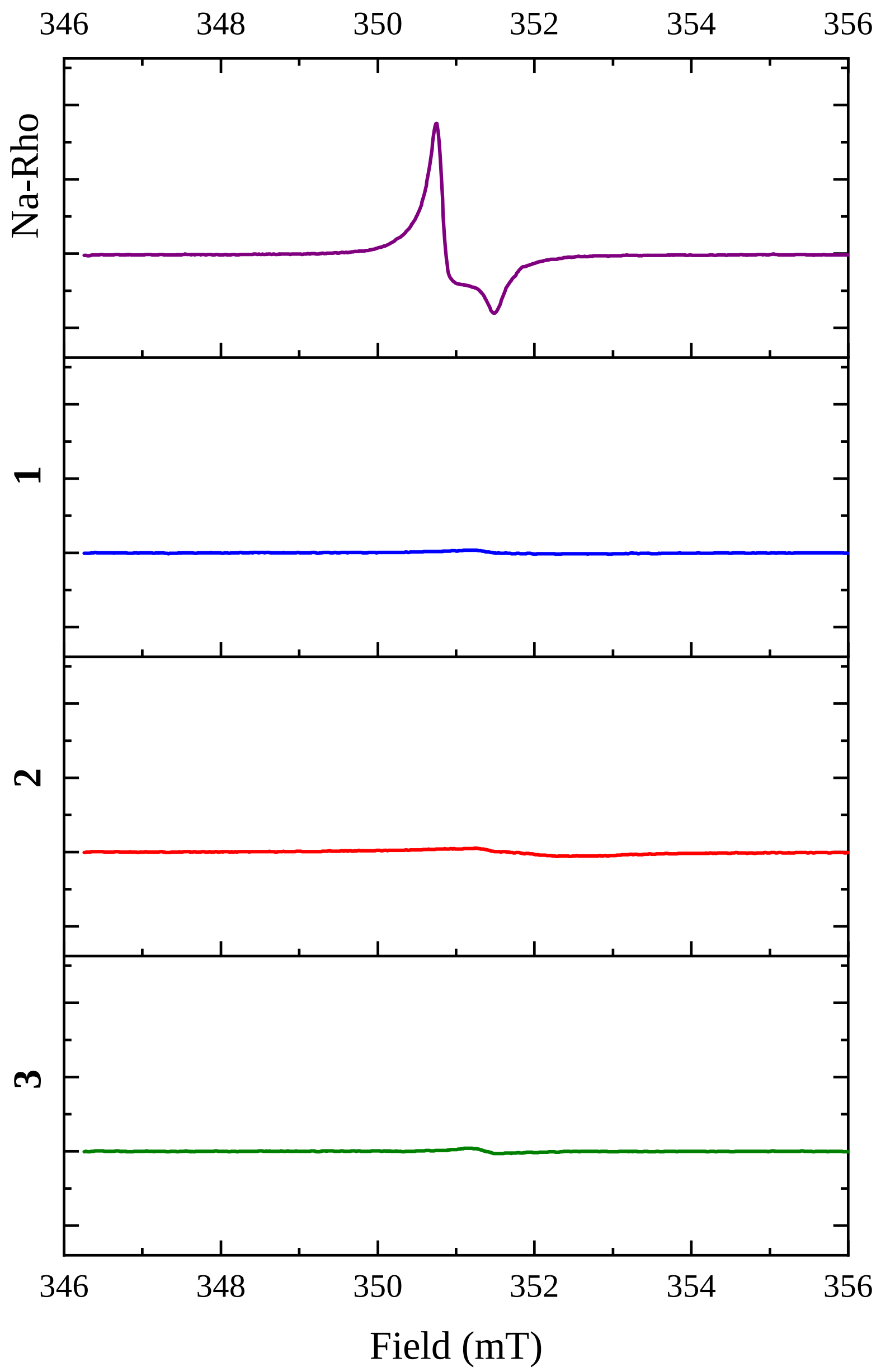

Figure S19: cwEPR with powder samples at 297 K, measured at 1.5 mW and plotted at 9.85 GHz.

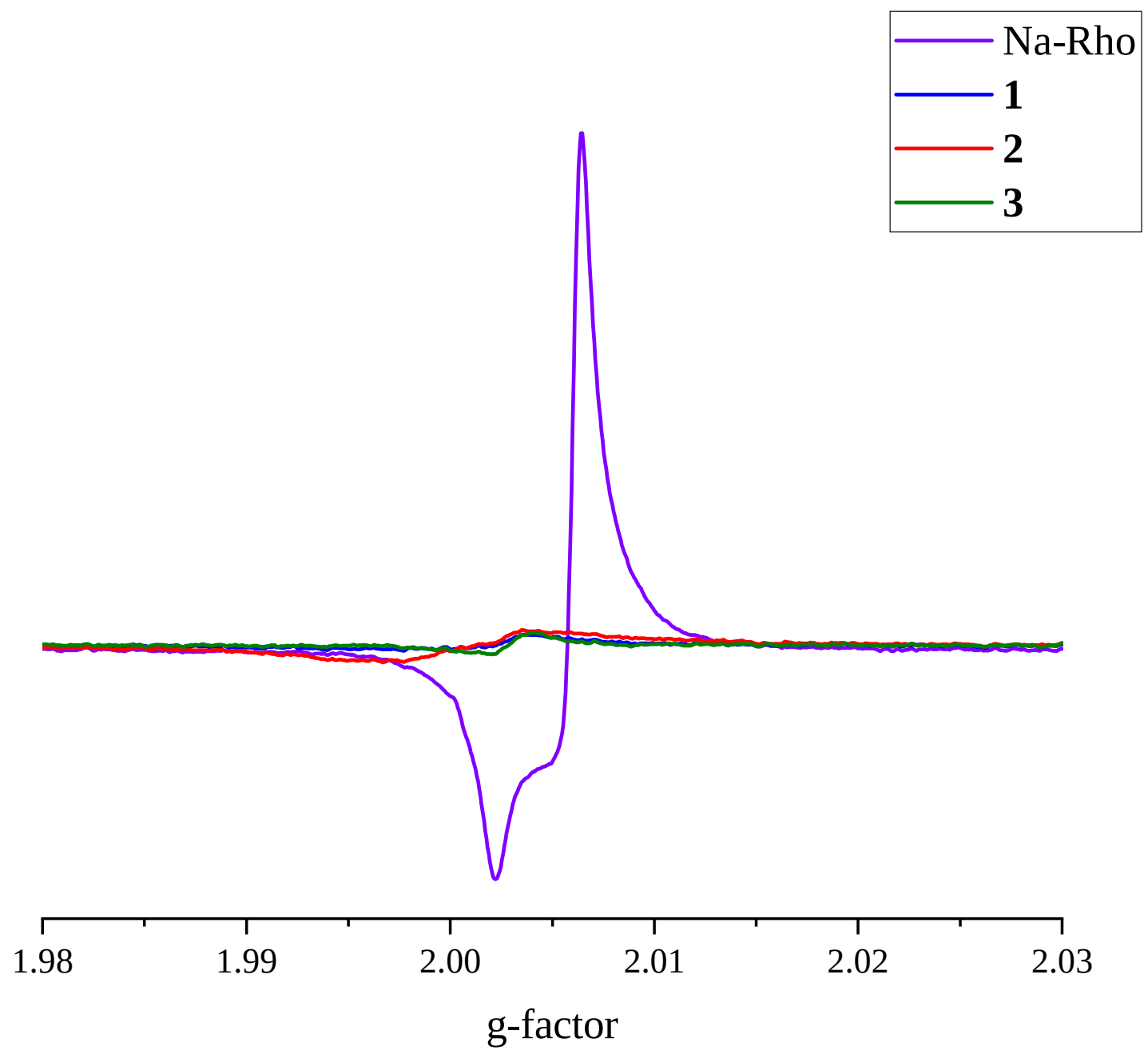

Figure S20: cwEPR with powder samples at 297 K, measured at 1.5 mW and plotted by g-factor.

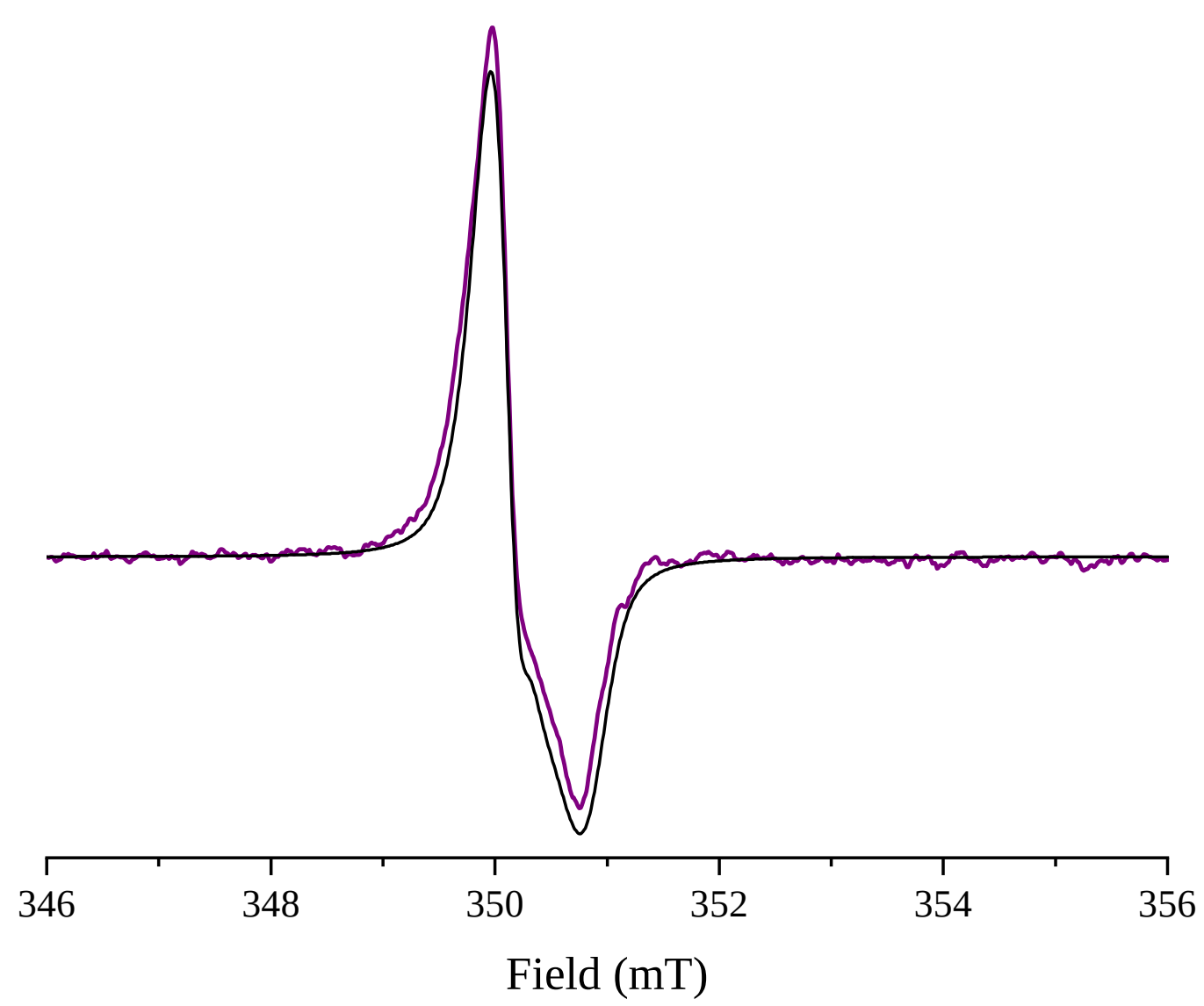

Figure S21: cwEPR with sodium rhodizonate at 60 K, measured at 0.0015 mW and plotted at 9.85 GHz.

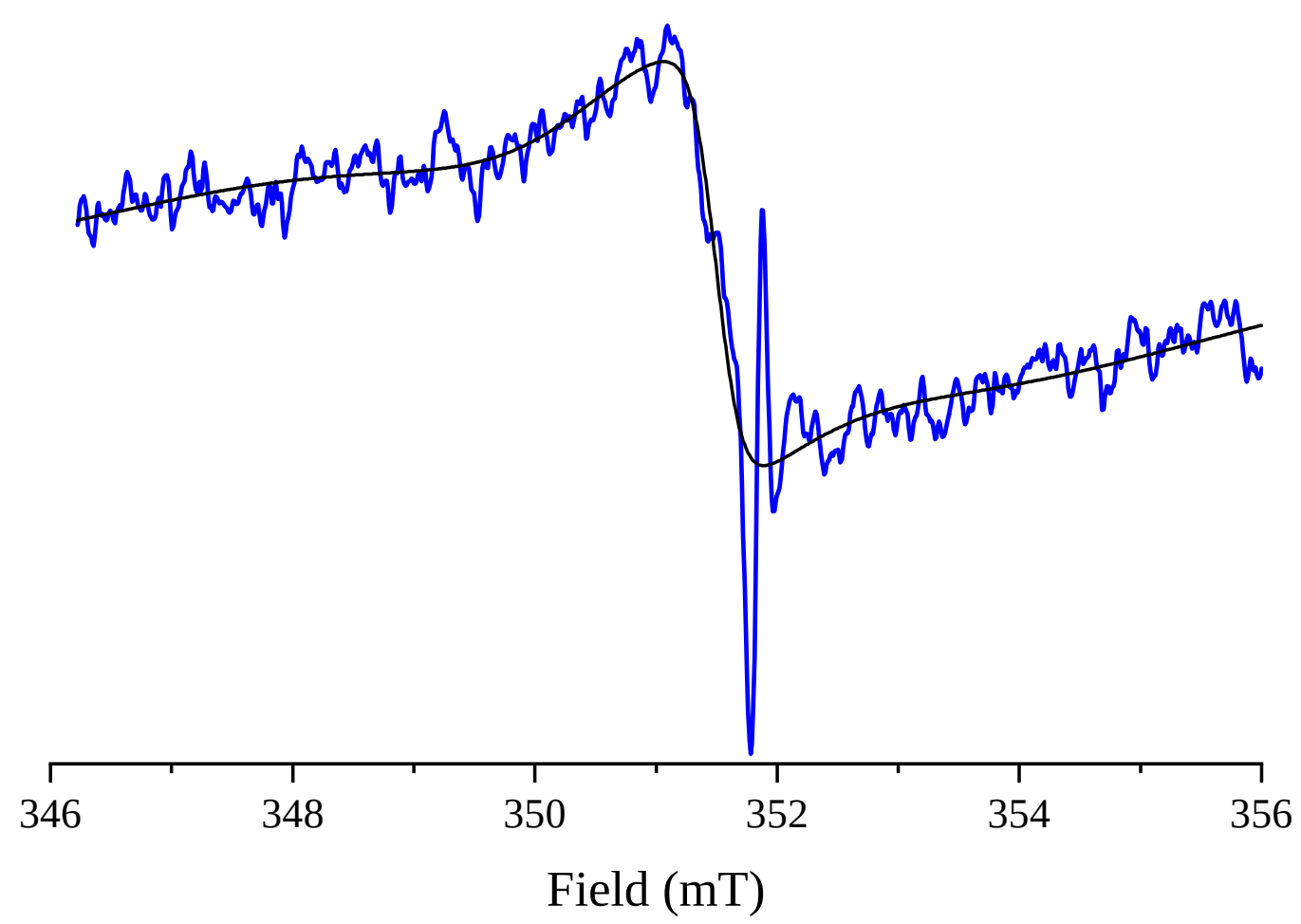

Figure S22: cwEPR with **1** at 60 K, measured at 0.0015 mW and plotted at 9.85 GHz.

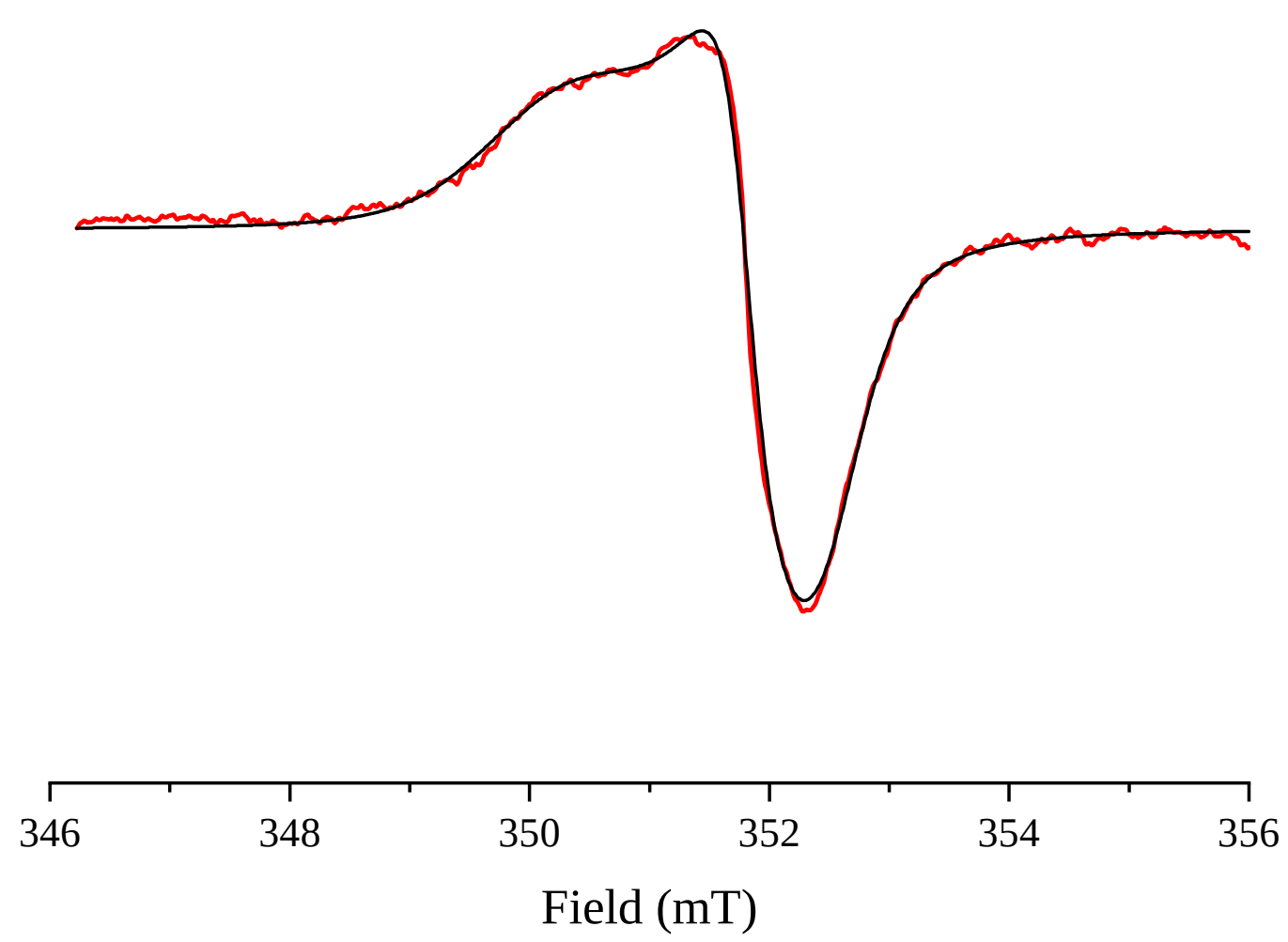

Figure S23: cwEPR with **2** at 60 K, measured at 0.0015 mW and plotted at 9.85 GHz.

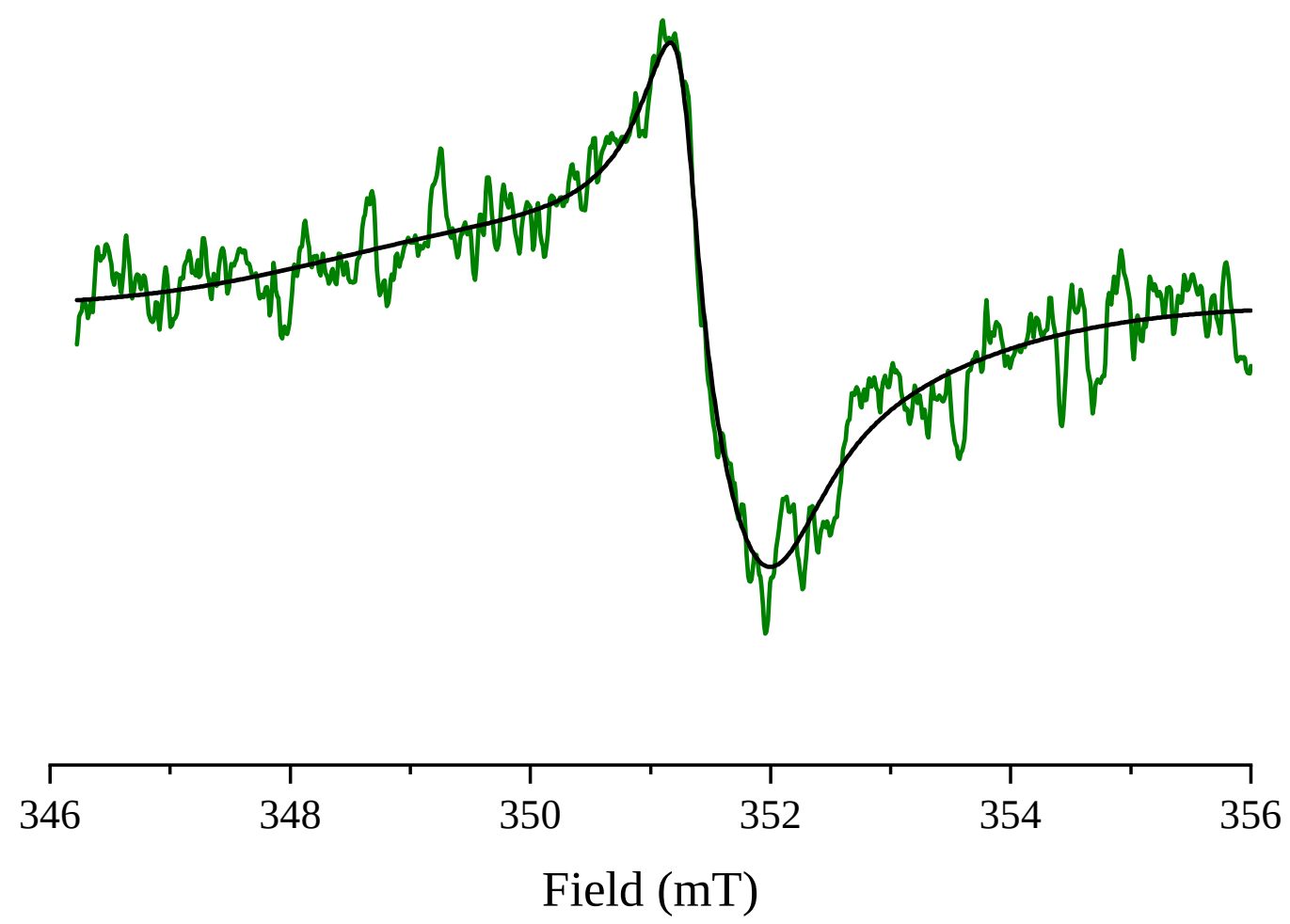

Figure S24: cwEPR with **3** at 60 K, measured at 0.0015 mW and plotted at 9.85 GHz.

5.1 Fitting

Datasets were analyzed using the EasySpin package for MATLAB.[9] Once the spectra were loaded, parameters for the anisotropic g-factor, g strain, and line widths were set depending on the sample spectra being fitted. The esfit and pepper functions were utilized to conduct the least squares fitting. The spectra were fitted with the Nelder-Mead simplex algorithm.

An example of EasySpin code used is shown below:

```
[B,spc,Param] = eprload('062525_RhoPbTartrate_60K_50dB_bs_bgsub.DSC');
Exp.mwFreq = 9.85e+09;
Exp.nPoints = numel(B);
mwcorrect = (0.1*Exp.mwFreq)/(9.35e+09);
B0 = (B*mwcorrect);
Exp.Range = [min(B0) max(B0)];
```

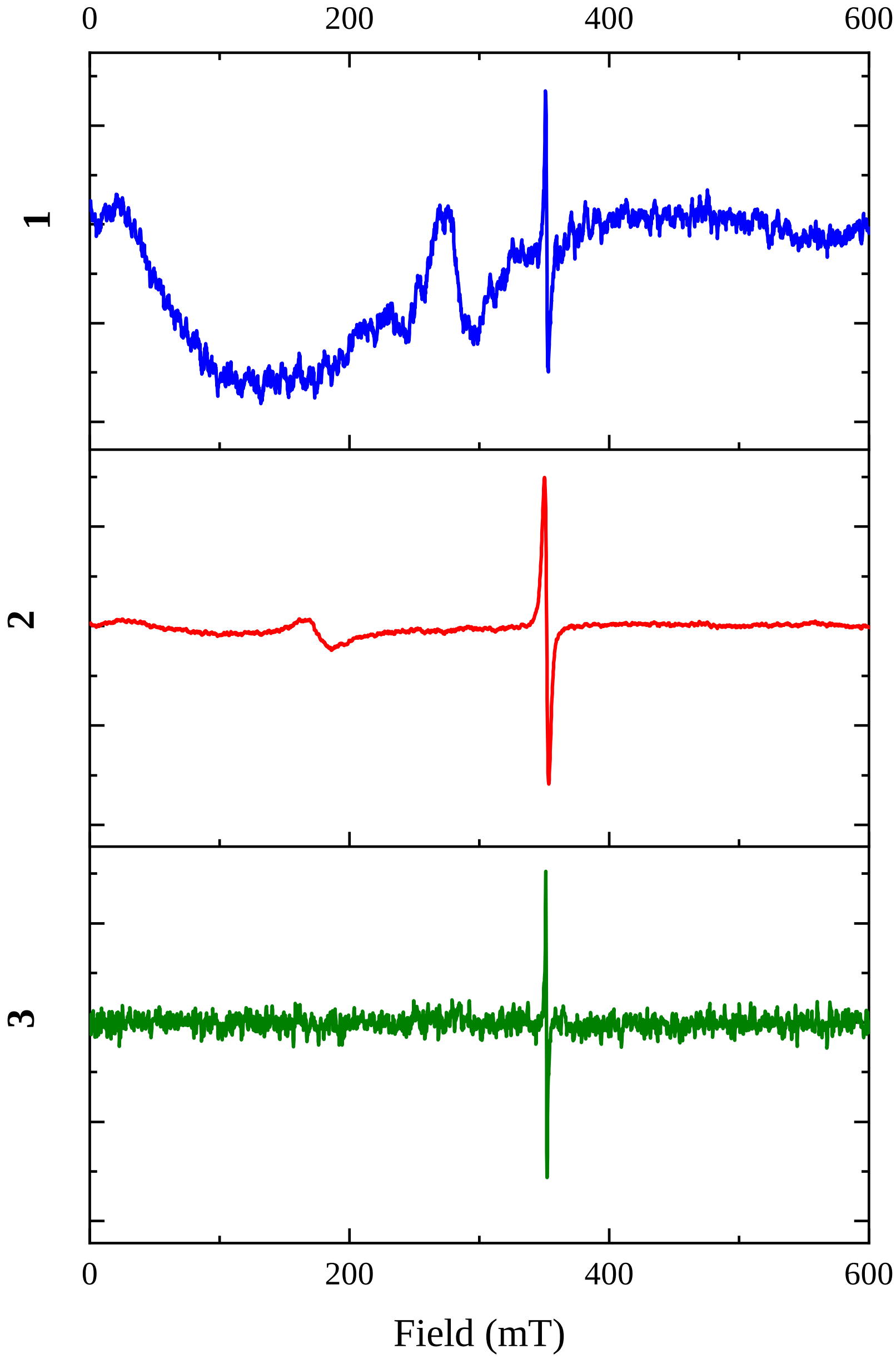

Figure S25: Room temperature cwEPR preliminary widescans, measured at 1.5 mW and plotted at 9.85 GHz. Broadening of rhodizonate signal observed due to 10 G modulation amplitude.

```
plot(B0,spc);
Exp.mwFreq = 9.85;
Sys.S = [1/2];
Sys.g = [2.0 2.001 2.02];
Sys.gStrain = [0.003 0.00 0.007];
Sys.lwpp = [0.041 0.36];
Sys.Nucs = '13C'
Sys.A = [4 8 18];
SysVary.g = [0.05 0.05 0.05];
%SysVary.gStrain = [0.05 0.05 0.05];
SysVary.lwpp = [0.05 0.05];
SysVary.A = [4 8 10]
Exp.mwFreq = 9.85;
Exp.Range = [min(B0) max(B0)];
esfit(spc,@pepper,{Sys,Exp},{SysVary});
%Least-squares fitting
esfit(spc,@pepper,{Sys,Exp},{SysVary});
```

References

(1) Silverman, J. A. *The Coordination Chemistry of the Rhodizonate Dianion and Its Decomposition Products*. Ph.D. thesis, Florida International University, 2021.

(2) Dinnebier, R. E.; Nuss, H.; Jansen, M. Disodium rhodizonate: a powder diffraction study. *Structure Reports* **2005**, *61*, m2148–m2150.

(3) Narayanan, A.; Hartman, J. S.; Bain, A. D. Characterizing nonexponential spin-lattice relaxation in solid-state NMR by fitting to the stretched exponential. *Journal of Magnetic Resonance, Series A* **1995**, *112*, 58–65.

(4) Morris, R. M.; Klabunde, K. J. Formation of paramagnetic adsorbed molecules on thermally activated magnesium and calcium oxides. Further studies of carbon monoxide. *Journal of the American Chemical Society* **1983**, *105*, 2633–2639.

(5) Giamello, E.; Murphy, D.; Marchese, L.; Martra, G.; Zecchina, A. Electron paramagnetic resonance investigation of the interaction of CO with the surface of electron-rich magnesium oxide: evidence for the CO–radical anion. *Journal of the Chemical Society, Faraday Transactions* **1993**, *89*, 3715–3722.

(6) Giannakopoulos, E.; Christoforidis, K.; Tsipis, A.; Jerzykiewicz, M.; Deligiannakis, Y. Influence of Pb (II) on the radical properties of humic substances and model compounds. *The Journal of Physical Chemistry A* **2005**, *109*, 2223–2232.

(7) Chechik, V.; Carter, E.; Murphy, D. *Electron Paramagnetic Resonance*; Oxford Chemistry Primers; Oxford University Press: Oxford, England, 2016.

(8) Tordo, P.; Bennati, M.; Lucarini, M.; Smirnov, A.; Stoll, S. *Electron Paramagnetic Resonance: Volume 22*; Royal Society of Chemistry, 2010; Vol. 22.

(9) Stoll, S.; Schweiger, A. EasySpin, a comprehensive software package for spectral simulation and analysis in EPR. *Journal of Magnetic Resonance* **2006**, *178*, 42–55.
